# CULLIN-3 Controls TIMELESS Oscillations in the *Drosophila* Circadian Clock

**DOI:** 10.1371/journal.pbio.1001367

**Published:** 2012-08-07

**Authors:** Brigitte Grima, Alexandre Dognon, Annie Lamouroux, Elisabeth Chélot, François Rouyer

**Affiliations:** 1 Institut de Neurobiologie Alfred Fessard, CNRS UPR3294, Gif-sur-Yvette, France; 2 Département de Biologie, Université Paris Sud, Orsay, France; University of Geneva, Switzerland

## Abstract

Eukaryotic circadian clocks rely on transcriptional feedback loops. In Drosophila, the PERIOD (PER) and TIMELESS (TIM) proteins accumulate during the night, inhibit the activity of the CLOCK (CLK)/CYCLE (CYC) transcriptional complex, and are degraded in the early morning. The control of PER and TIM oscillations largely depends on post-translational mechanisms. They involve both light-dependent and light-independent pathways that rely on the phosphorylation, ubiquitination, and proteasomal degradation of the clock proteins. SLMB, which is part of a CULLIN-1-based E3 ubiquitin ligase complex, is required for the circadian degradation of phosphorylated PER. We show here that CULLIN-3 (CUL-3) is required for the circadian control of PER and TIM oscillations. Expression of either *Cul-3* RNAi or dominant negative forms of CUL-3 in the clock neurons alters locomotor behavior and dampens PER and TIM oscillations in light-dark cycles. In constant conditions, CUL-3 deregulation induces behavioral arrhythmicity and rapidly abolishes TIM cycling, with slower effects on PER. CUL-3 affects TIM accumulation more strongly in the absence of PER and forms protein complexes with hypo-phosphorylated TIM. In contrast, SLMB affects TIM more strongly in the presence of PER and preferentially associates with phosphorylated TIM. CUL-3 and SLMB show additive effects on TIM and PER, suggesting different roles for the two ubiquitination complexes on PER and TIM cycling. This work thus shows that CUL-3 is a new component of the Drosophila clock, which plays an important role in the control of TIM oscillations.

## Abstract 2

The ubiquitin ligases CUL-3 and SLMB collaborate to regulate the *Drosophila* circadian clock by controlling TIMELESS oscillations.

## Author Summary

Circadian clocks adjust the physiology and behavior of organisms to the day/night cycle and rely on molecular feedback loops that generate daily oscillations of transcription. In the *Drosophila* fruit fly, the PERIOD (PER) and TIMELESS (TIM) proteins coordinate the clock–they accumulate during the night, form a complex, and repress their own gene expression in the early morning. The temporal control of this oscillation involves the phosphorylation, ubiquitination, and proteasomal degradation of the PER and TIM proteins. The SUPERNUMERARY LIMBS (SLMB) ubiquitin ligase is known to play a key role in controlling the degradation of phosphorylated PER and TIM. In this study we investigated the role of another ubiquitin ligase, CULLIN-3 (CUL-3). We found that inhibition of CUL-3 activity results in the abolition of rest/activity rhythms in flies and flattens the PER and TIM oscillations. CUL-3 physically interacts and forms a complex with a lowphosphorylated version of TIM in the absence of PER, thereby allowing its accumulation during the night. In contrast, when PER is present SLMB preferentially interacts with phosphorylated TIM, favoring its degradation. The results suggest that CUL-3 and SLMB share the work to control the oscillations of the PER and TIM proteins during the day/night cycle.

## Introduction

Circadian clocks are present in most living organisms and control a variety of physiological and behavioral functions. Eukaryotic clocks stem from a transcriptional negative feedback loop where activators induce the expression of repressors, which then inhibit the activators [1]. The accumulation of the repressor proteins is delayed by post-translational mechanisms, allowing us to define active and inactive phases of transcription, hence an oscillation. In Drosophila, the two basic helix-loop-helix PER-ARNT-SIM (bHLH PAS) proteins CLOCK (CLK) and CYCLE (CYC) activate the transcription of their targets in the evening [2],[3]. The target genes *period* (*per*) and *timeless* (*tim*) encode two proteins that associate in a complex, progressively accumulate, and become phosphorylated to repress CLK/CYC-dependent transcription in the late night. The delayed accumulation, nuclear entry, and transcriptional activity of PER and TIM involve their phosphorylation by several kinases such as DOUBLE TIME (DBT) CK1ε [4]–[10] and NEMO [11] that target PER. The CK2 casein kinase [12]–[14] and SHAGGY (SGG) GSK3 [15] rather target TIM. The phosphorylation of PER and TIM are also regulated by the phosphatases PP2A [16] and PP1 [17], respectively. Nuclear phosphorylated PER induces CLK phosphorylation and removal from the chromatin, then PER degradation in the morning allows CLK-CYC-dependent transcription to resume [18]–[21].

The pace of the oscillation depends largely on the speed of PER/TIM accumulation during the early night and degradation in the late night. The stability of phosphorylated PER and TIM is controlled by the SUPERNUMERARY LIMBS (SLMB) E3 ubiquitin ligase [22],[23]. For PER, the phosphorylation of a few serine residues around S47 by DBT cooperatively increases SLMB binding to PER [24] and is negatively regulated by NEMO-controlled phosphorylation in the PER^S^ region [11] to finely tune PER stability. Both S47 phosphorylation and direct SLMB-binding occur at the end of the night, suggesting that SLMB controls nuclear PER at the end of the cycle [24]. DBT has been proposed to also induce cytoplasmic PER degradation in the absence of TIM and thus control the delayed accumulation of PER [4],[6]. This model predicts that the control of cytoplasmic PER stability depends on TIM accumulation. CK2 has been suggested to destabilize TIM [14], whereas PP1 appears to favor TIM stabilization [17], but whether they affect SLMB function is unknown.

In addition to SLMB that controls the circadian oscillations of TIM, the JETLAG (JET) E3 ligase targets TIM and CRYPTOCHROME (CRY) for the light-induced proteasome-dependent protein degradation that participates to the resetting of the clock [25]–[27]. The JET-dependent degradation, but not the SLMB-dependent one, appears to be regulated by the COP9 signalosome [28]. SLMB and JET are parts of CULLIN-1-based SCF complexes that belong to the RING family of E3 ubiquitin ligases [29]. SLMB plays a major role in CUBITUS INTERRUPTUS (CI) proteolysis, which generates the repressor form of the CI transcription factor in the absence of HEDGEHOG (HH) signaling [30]. In addition to SLMB, other components of the HH pathway such as CK1, CK2, and SGG are shared with the circadian oscillator [31]. Another ubiquitin complex, based on CULLIN-3, has been shown to participate in the proteolytic regulation of CI [32]–[35]. Since SLMB alone unlikely regulates all aspects of the circadian control of PER and TIM stability, we asked whether CULLIN-3 may participate in the control of PER and/or TIM oscillations. We show that CUL-3 downregulation induces strong defects of rest-activity rhythms, which mainly result from the loss of TIM cycling. Indeed, CUL-3 forms protein complexes with hypo-phosphorylated TIM, which are favored in the absence of PER, suggesting that CUL-3 participates in the control of the night accumulation of TIM. In contrast, SLMB is present in complexes that contain more phosphorylated TIM and are favored by the presence of PER, suggesting a rather later role in the TIM cycle.

## Results

### Deregulation of CUL-3 in the Clock Neurons Induces Behavioral Arrhythmicity

**Figure 1:**
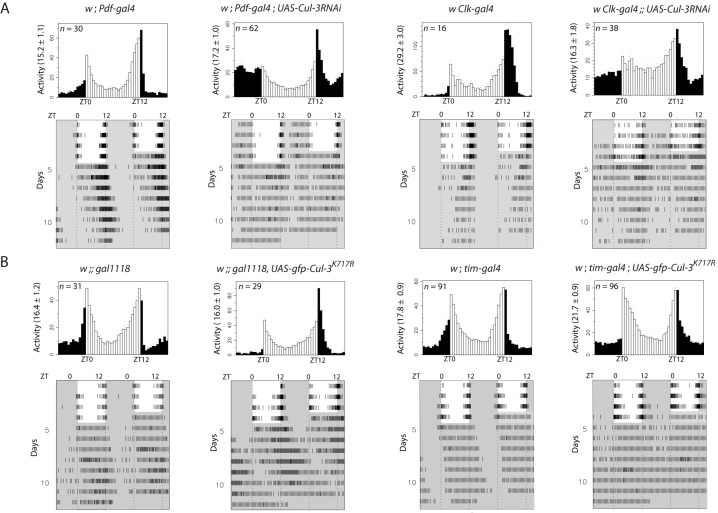
Locomotor activity of *Cul-3* downregulated flies in LD and DD. Control flies and flies expressing either a *Cul-3* RNAi (A) or a CUL-3^K717R^ protein (B) were entrained for 4 d in LD 12∶12 and transferred to DD. White and black/gray indicate lights-ON and lights-OFF, respectively. ZT is Zeitgeber Time (ZT0 corresponds to lights-ON). Top panels: averaged activity distribution of *n* flies in LD (see Materials and Methods). Dots indicate the s.e.m. of the activity for each 0.5-h interval. Average activity per 0.5-h is indicated in parentheses on the left. Bottom panels: averaged actograms during both LD and DD conditions (see Materials and Methods). Behavioral analyses were repeated three to four times with very similar results.

Since *Cul-3* mutants are homozygous lethal, we first used targeted expression of *Cul-3* RNAi to test the possible role of CUL-3 in the control of behavioral rhythms. About 150 neurons express PER and TIM in the brain, and various studies have assigned specific behavioral contributions to defined neuronal subsets, depending on the environmental conditions [36]. In particular, PER cycling in the lateral neurons (LNs) that express the Pigment-Dispersing Factor (PDF) generates morning activity in light-dark (LD) cycles and free running rhythms in constant darkness (DD), whereas PER cycling in the PDF-negative LNs generates LD evening activity [37],[38]. Expressing *Cul-3* RNAi in all clock cells under *tim-gal4* control induced 100% lethality, but a limited number of adult flies could be obtained with the *Clk-gal4* driver, whose expression is less broad than *tim-gal4* [39],[40], and no lethality was observed with the more restricted *cry-gal4* and *Pdf-gal4* drivers. In LD cycles, flies expressing *Cul-3* RNAi under *Pdf-gal4* or *Clk-gal4* control displayed no or reduced morning anticipatory activity (Figure 1A and Table S1). The two genotypes also showed reduced lights-ON startle response. After transfer to DD, RNAi genotypes either showed weak rhythms or became arrhythmic (Figure 1A and Table 1). We also used the *gal1118* driver, which is mostly expressed in the PDF cells [41]. Reduced morning anticipation in LD and strongly altered rhythms in DD were observed when *Cul-3* RNAi expression was driven by *gal1118* (Figure S1). No disappearance of the lights-ON startle response was observed, supporting a genetic background effect in the previous genotypes. Importantly, the effect of *Cul-3* RNAi on *Cul-3* mRNA levels was tested by quantitative RT-PCR (Figure S2). *Cul-3RNAi* induced a 2-fold decrease of *Cul-3* mRNAs in head extracts when driven by *Clk-gal4* or 5-fold in larvae when using the ubiquitous *da-gal4* (*daughterless*), supporting decreased CUL-3 activity in the RNAi flies. For RNAi genotypes, two copies of both the *gal4* and *UAS* transgenes were required for generating arrhythmic behavior (Figures 1A and S1, and Table 1).

**Table 1:** Locomotor activity rhythms in constant darkness of flies with altered CUL-3 activity.

Genotype	Total Flies	Rhythmic Flies (%)	Period (h)	Power
*w ;; UAS-Cul-3RNAi*	27	93	24.1±0.1	154±14
*w ; Pdf-gal4 ; UAS-Cul-3RNAi*	47	55	25.1±1.2	57±5
*w ; Pdf-gal4*	28	100	25.0±0.2	145±10
*w ; cry-gal4-39 ; UAS-Cul-3RNAi*	28	30	26.5±2.0	43±5
*w ; cry-gal4-39*	20	90	24.6±0.1	159±7
*w Clk-gal4 ;; UAS-Cul-3RNAi*	26	61	24.1±0.7	55±7
*w Clk-gal4*	16	94	23.7±0.1	117±15
*w ;; gal1118, UAS-Cul-3RNAi*	25	44	23.3±0.4	74±9
*w ;; gal1118*	29	100	24.5±0.1	118±10
*w ;; UAS-gfp-Cul-3K717R*	27	100	23.7±0.1	173±12
*w ;; gal1118, UAS-gfp-Cul-3K717R*	29	72	27.1±1.0	75±9
*w ; tim-gal4 ; UAS-gfp-Cul-3K717R*	96	40	26.8±0.9	56±7
*w ; tim-gal4 ; cry-gal80, UAS-gfp-Cul-3K717R/UAS-gfp-Cul-3K717R*	37	97	24.1±0.1	123±8
*w ;; UAS-flag-Cul-3K717R/+*	32	100	23.3±0.1	169±12
*w ; tim-gal4 ; UAS-flag-Cul-3K717R/+*	23	17	27.4±2.9	31±5
*w ;; UAS-Cul-3ΔC*	30	93	24.3±0.1	174±11
*w ; tim-gal4 ; UAS-Cul-3ΔC*	48	29	25.4±0.7	73±12
*w ;; UAS-gfp-Cul-3K717R/UAS-Cul-3ΔC*	32	97	24.0±0.2	113±11
*w ; tim-gal4 ; UAS-gfp-Cul-3K717R/UAS-Cul-3ΔC*	54	9	28.3±3.8	49±20
*w ;; UAS-gfp-Cul-3*	40	100	24.3±0.1	178±10
*w ; tim-gal4 ; UAS-gfp-Cul-3*	25	36	25.4±1.6	53±10
*w ; tim-gal4*	96	89	25.3±0.2	113±7
*w ; Pdf-gal4 ; UAS-Cul-3RNAi (20°C)*	61	82	23.9±0.4	91±8
*w ; Pdf-gal4 (20°C)*	30	100	24.6±0.2	131±9

The mean values of period and associated power (see Materials and Methods) are given ± s.e.m. In genotypes with altered CUL-3 activity, rhythmic flies display weak rhythms as indicated by the large s.e.m. of the period and the low associated power.

Two inactive forms of CUL-3 were then expressed in the clock neurons. Both forms lack the site for the addition of Nedd8, a CULLIN modification that is required for ubiquitin transfer from E2 to the substrate [42]. CUL-3^K717R^ bears a mutation in the neddylation site [43], whereas CUL-3^ΔC^ lacks the conserved C-terminal domain that includes this site [44]. Expression of one or the other inactive protein under the control of a *Pdf-gal4*, *cry-gal4*, or *Clk-gal4* driver did not significantly affect behavior (unpublished data), suggesting that they were less efficient than RNAi for decreasing CUL-3 activity in the clock neurons. Driving their expression with *tim-gal4* did not induce lethality, also supporting weaker effect compared to *Cul-3* RNAi. We thus used *gal1118* and *tim-gal4* to test the effect of CUL3^K717R^ on behavioral rhythms. LD morning anticipation was reduced and DD free running rhythms were lost or altered in flies expressing either one of two different CUL3^K717R^-encoding constructs (Figures 1B and S3, Tables 1 and S1), indicating that the modified CUL-3 protein was acting as a dominant negative form. Rhythms were restored when GAL4 activity was inhibited by a *cry-gal80* transgene, confirming that CUL-3 was required in clock cells for controlling behavior (Table 1). Altered morning anticipation in LD (Figure S3, Table S1, and unpublished data) and behavioral arrhythmicity in DD (Figure S3 and Table 1) were also observed in flies expressing CUL-3^ΔC^, both CUL3^K717R^ and CUL3^ΔC^, or wild-type CUL-3, under *tim-gal4* control. The latter genotype suggested that supernumerary CUL-3 molecules might interfere with the function of the protein, as reported for SLMB [22]. Targeted expression of RNAi or mutant protein as well as overexpression of the wild type protein thus revealed that CUL-3 activity was required in the clock neurons for strong morning anticipation in LD and rhythmic behavior in DD. Although *tim-gal4* and *Clk-gal4* are expressed in all clock neurons, evening anticipation could still be observed in flies with altered CUL-3 activity.

Since *Cul-3* mutants affect the axonal growth of the mushroom body neurons [43], we tested the possibility that developmental effects of CUL3 deregulation would be responsible for the behavioral defects. We took advantage of the fact that GAL4-driven expression is temperature-dependent [45] and compared the behavior of RNAi flies grown at 25°C and tested at either 25°C or 20°C. Adult flies tested at 20°C showed significantly weaker defects than those tested at 25°C (Figure S4 and Table 1), indicating that the adult stage was determinant for CUL-3 function in activity rhythms. We also checked the morphology of the PDF cells expressing *Cul-3* RNAi or inactive CUL-3 proteins (Figure S5). Anti-PDF labeling did not show any detectable morphological defects of the PDF-positive ventral lateral neurons in flies expressing *Cul-3* RNAi. Slightly more defasciculated projections and reduced arborization in the medulla were observed for flies expressing CUL-3^ΔC^ but not in the other genotypes. One or two additional PDF-positive cells were sometimes seen in flies expressing either CUL-3^ΔC^ or CUL-3^K717R^ (unpublished data). Altogether, the data indicated that the behavioral phenotype induced by CUL-3 downregulation was, at least for a large part, not a consequence of a developmental defect.

### CUL-3 Controls PER and TIM Oscillations in the Clock Neurons

**Figure 2:**
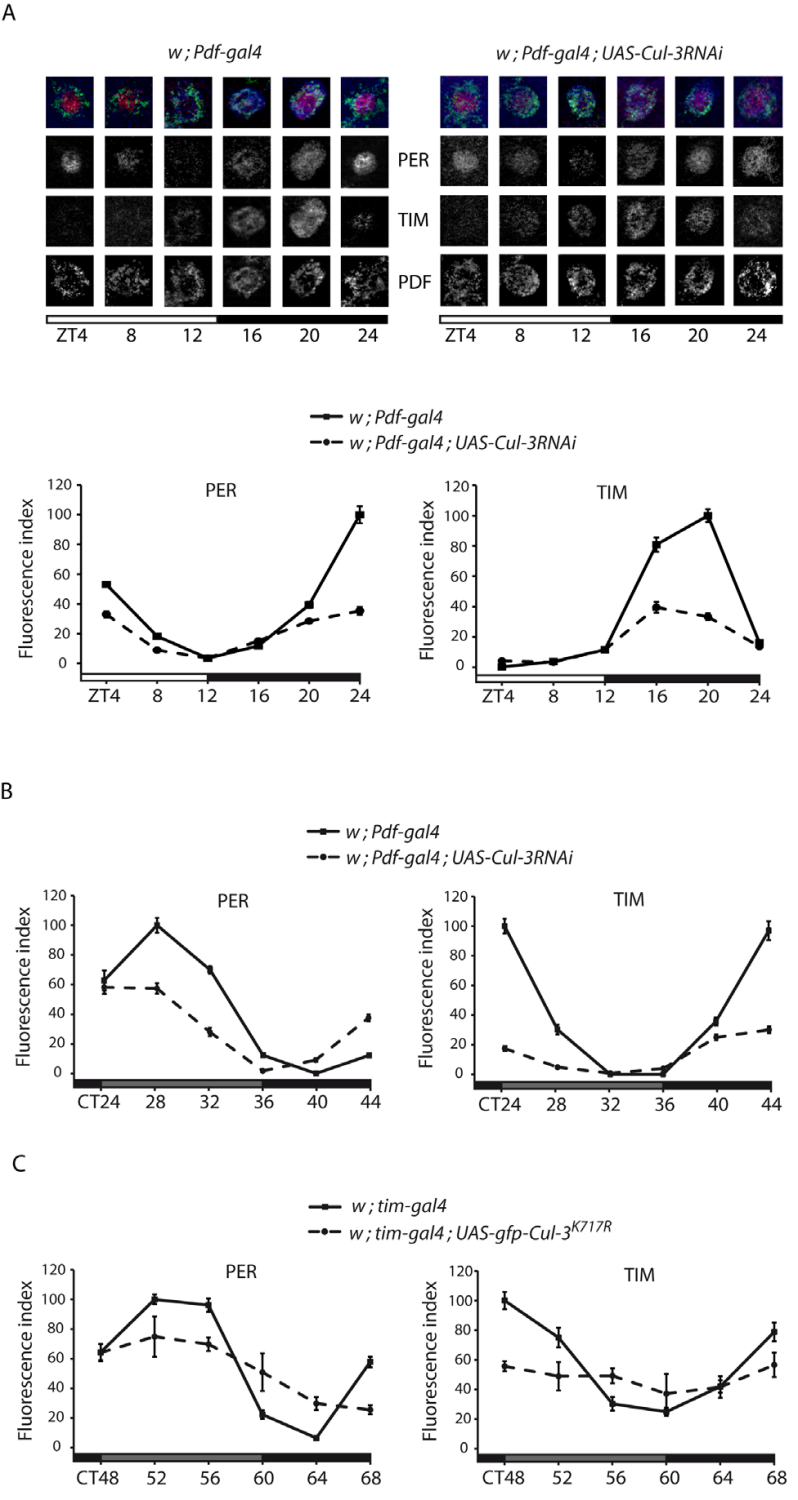
PER and TIM in the s-LNvs of flies expressing *Cul-3* RNAi. Flies were entrained for 3 d in LD and collected every 4 h on the fourth day of LD (A) or transferred to DD and collected on the second (B) or third (C) day of DD. Images are optical sections of individual PDF-expressing s-LNvs from *Pdf>RNAi* flies and controls in LD. Merged images: red is PER, blue is TIM, and green is PDF. Graphs represent quantifications of PER and TIM immunolabeling (see Materials and Methods) in the PDF-positive s-LNvs of *Pdf>RNAi* or *tim>Cul-3^K717R^* flies and relevant controls. White and gray bars indicate lights-ON and lights-OFF, respectively. Gray and black bars indicate subjective day and subjective night, respectively. Time (h) is indicated as ZT or CT (Circadian Time) where CT0 is 12 h after lights-OFF of the last LD day. Error bars indicate s.e.m. Two independent experiments were done for each genotype/condition with very similar results.

To understand the molecular bases of the behavioral alterations displayed by the flies with deregulated CUL-3, PER and TIM oscillations were analyzed in their PDF-expressing small ventral lateral neurons (s-LNvs). In LD conditions, *w ; Pdf-gal4; UAS-Cul-3RNAi* (hereafter *Pdf>RNAi*) flies showed dampened PER and TIM oscillations (Figure 2A). The two proteins failed to reach wild-type peak levels during the night, indicating that their nighttime accumulation was affected by *Cul-3* downregulation. Interestingly, PER and TIM appeared slightly more evenly distributed between nuclear and cytoplasmic compartments in the RNAi flies (see ZT24 in particular). Similarly dampened oscillations of the two proteins were also observed in the second day after transfer in DD (Figure 2B), providing a molecular basis for the behavioral arrhythmicity of these flies. Arrhythmic *w ; tim-gal4 ; UAS-gfp-Cul-3^K717R^* (*tim>Cul-3^K717R^*) flies also showed altered PER and TIM oscillations, with a complete loss of TIM cycling on the third day of DD (Figure 2C). Although these flies should express inactive CUL-3 in all clock cells, they still displayed evening activity in LD conditions (see above). We thus suspected that PER and TIM oscillations might not be affected in their “evening” CRY-expressing dorsal lateral neurons (LNds). Indeed, a 30%–40% decrease of TIM immunoreactivity was observed at peak time (ZT16-20) in the s-LNvs of *tim>Cul-3^K717R^* flies, whereas mutants and controls showed similar TIM labeling in the CRY-positive LNds (Figure S6).

### CUL-3 Acts on PER and TIM Post-Transcriptionally

**Figure 3:**
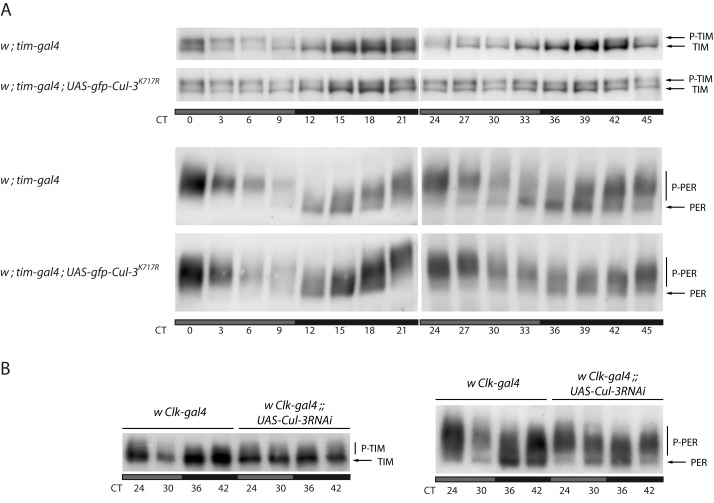
PER and TIM in head extracts of flies expressing CUL-3^K717R^ or *Cul-3* RNAi. Flies were entrained for 3 d in LD, then transferred to DD. Gray and black bars indicate subjective day and subjective night, respectively. (A) TIM (top, Tris-acetate gel) and PER (bottom, Tris-glycine gel) Western blot analysis of head extracts from flies collected the first and second days of DD. Phosphorylated (P-) and hypo-phosphorylated forms of PER and TIM are indicated. (B) TIM (left) and PER (right) Western blots (both Tris-glycine gels) of head extracts from flies collected the second day of DD. Two (*Cul-3RNAi*) or three (*Cul-3^K717R^*) independent Western blots were done for each condition with very similar results.

PER and TIM oscillations were analyzed in head extracts of *tim>Cul-3^K717R^* flies. In LD conditions, mutant flies still showed robust PER (unpublished data) and TIM (Figure S7) cycling. Since a strong dampening of PER and TIM oscillations was observed in the PDF cells under the same conditions, it suggested CUL-3 might have a less crucial role in the eye, which is the major source of clock proteins in the head [46], at least in LD cycles. Alternatively, the lower expression of the *tim-gal4* driver in the eye (unpublished data) might not produce sufficiently high levels of CUL-3^K717R^ protein to affect LD oscillations, which are strongly driven by light. We then looked at protein oscillations in DD (Figure 3A and Figure S8). At DD1, only subtle defects could be observed on PER and TIM cycling, with slightly more phosphorylated proteins at night in the mutant. At DD2, TIM cycling was strongly altered in the mutant with similar levels of phosphorylated TIM at all time points and weak cycling of unphosphorylated TIM, which stayed at relatively low levels. A very similar phenotype was observed in flies with two copies of *tim-gal4* driving a single copy of *UAS-flag-Cul-3^K717R^* (Figure S9) or a single copy of both *UAS-gfp-Cul-3^K717R^* and *UAS-Cul-3^ΔC^* (unpublished data). Phosphatase treatment abolished the slow migrating TIM forms of *tim>Cul-3^K717R^* flies (Figure S10), demonstrating that they were indeed phosphorylated TIM. The constitutive presence of both phosphorylated and unphosphorylated forms of TIM supported a post-translational effect of CUL-3. Similarly altered TIM cycling was observed in flies with overexpressing wild type CUL-3 (Figure S11), suggesting that excess of wild type protein might interfere with the formation of functional complexes with the proper stoichiometry. Since *tim>Cul-3RNAi* were lethal, we analyzed TIM in head extracts of *Clk>Cul-3RNAi* flies, although it is less efficient than *tim-gal4* to affect molecular oscillations in head extracts when used to drive RNAi targeted against various clock genes (unpublished data). At DD2, TIM cycling was strongly reduced, but the accumulation of phosphorylated TIM was less prominent than in flies expressing CUL-3^K717R^ (Figure 3B). The data indicated that deregulating CUL-3 strongly reduces TIM cycling, with mostly hypo-phosphorylated TIM in *Clk>Cul-3RNAi* flies and similar amounts of phosphorylated TIM and hypo-phosphorylated TIM in *tim>Cul-3^K717R^* flies.

In both *tim>Cul-3^K717R^* and *Clk>Cul-3RNAi* flies, PER cycling was still observed at day 2, but it was dampened. More phosphorylated and less unphosphorylated protein was observed at the beginning of the night (CT36), with a stronger phenotype in the *tim>Cul-3^K717R^* flies (Figure 3A,B). Since PER oscillations were less rapidly affected than TIM oscillations, it suggested that CUL-3 might control TIM more directly than PER.

**Figure 4:**
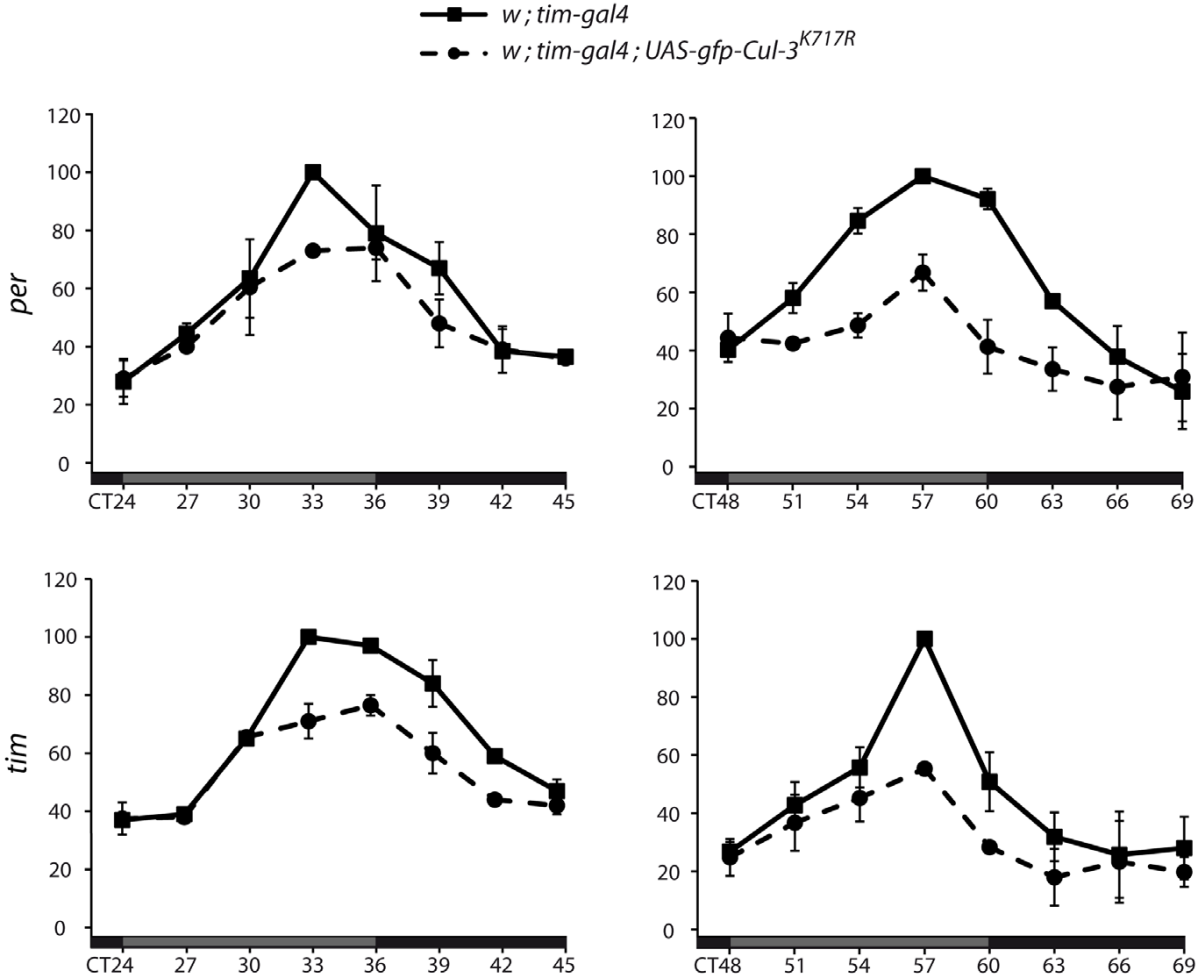
*per* and *tim* mRNA oscillations in flies expressing CUL-3^K717R^. Flies were entrained for 3 d in LD and transferred to DD for collection every 3 h in the second (left) and third (right) days of DD. Relative *per* and *tim* mRNA levels in head extracts were determined by quantitative RT-PCR (see Materials and Methods). For each experiment, the averaged normalized values of both mutant and control genotypes were expressed as a percentage of the maximum value set to 100. Means of two independent experiments are reported in the graphs. Error bars indicate the range between the two experimental values.

We then asked how CUL-3 deregulation affected *per* and *tim* mRNA levels. mRNA were quantified in head extracts of *tim>Cul-3^K717R^* and control flies (Figure 4). For both *per* and *tim*, mRNA levels still cycled at day 2 (left panels) in the mutant, but peak levels were about 25% lower. At day 3 (right panels), *per* and *tim* mRNA oscillations were more severely dampened, with peak levels lowered by about 40%. Decreased mRNA thus likely contributed to the lower levels of unphosphorylated PER and TIM at the beginning of the night (see Figure 3A). Since *tim* mRNA levels still showed oscillations at DD2 while phosphorylated TIM was already flat, it was unlikely that the protein defect was a consequence of altered mRNA cycling. PER protein and *per* mRNA showed dampened oscillations at DD2 in the flies expressing CUL-3^K717R^. However, the mRNA increase began to slow down at CT30 when more phosphorylated PER was observed (see Figure 3A), suggesting that higher phosphorylated PER levels were responsible for lower mRNA accumulation and subsequent lower PER and TIM synthesis. The data thus supported CUL3 acting at the protein level to control PER and TIM oscillations.

### The Control of Phosphorylated TIM by CUL-3 Does Not Require PER

**Figure 5:**
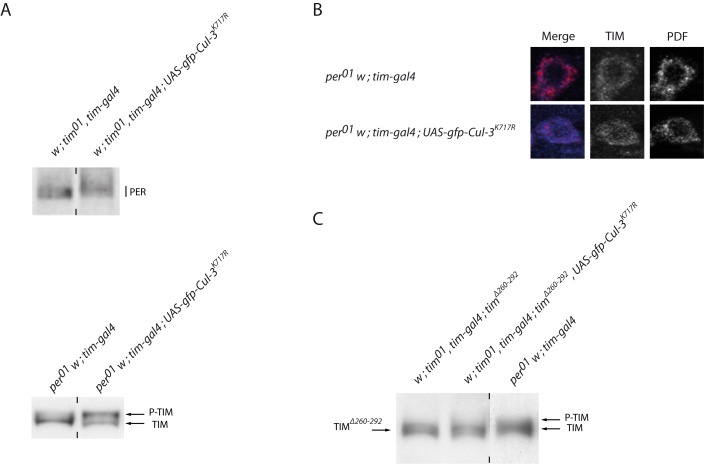
PER and TIM proteins in *per* or *tim* mutants expressing CUL-3^K717R^. Flies were entrained for 3 d in LD, transferred to DD, and collected at CT27. (A) anti-PER (top) and anti-TIM (bottom) Western blots of head extracts in a *tim^0^* and *per^0^* backgrounds, respectively. (B) Optical section of an individual s-LNv immunolabeled for TIM (blue) and PDF (red) in a *per^0^* background. (C) Anti-TIM Western blot of head extract in a *tim^0^ tim^Δ260–292^* background. Vertical dashes indicate that the two surrounding slots were not contiguous on the gel (A) or that a different exposure was used (C) (shorter exposure for *per^0^* extracts, which have more TIM). At least two independent experiments were done for each assay with very similar results.

**Figure 6:**
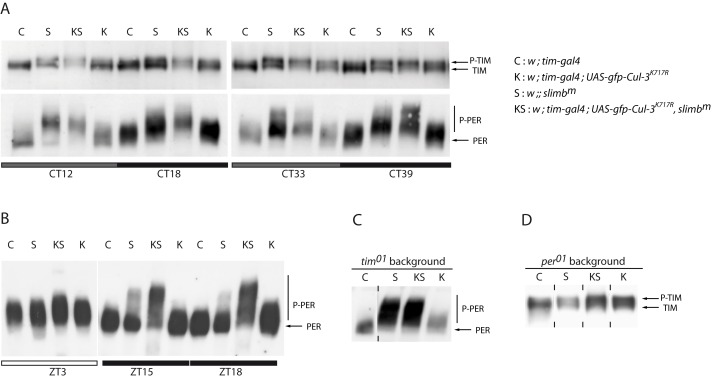
PER and TIM proteins in flies *Cul-3^K717R^* and *slmb^m^* flies. (A) Anti-TIM (top) and anti-PER (bottom) Western blots of head extracts. Flies were entrained 3 d in LD and transferred to DD for collection during the first and second day of DD. Gray and black bars indicate subjective day and subjective night, respectively. (B) Anti-PER Western blots of head extracts. Flies were entrained 3 d in LD and collected the fourth day. White and black bars indicate day and night, respectively. (C, D) Anti-PER (C) and anti-TIM (D) Western blots of head extracts *tim^0^* and *per^0^* backgrounds, respectively. Flies were entrained for 3 d in LD and transferred to DD for collection at CT27. Vertical dashes indicate that the two surrounding slots were not contiguous on the gel. Two to three independent Western blots were done for each condition with very similar results.

To understand how CUL-3 controls PER and TIM proteins, we analyzed PER in *tim^0^ tim>Cul-3^K717R^* flies and TIM in *per^0^ tim>Cul-3^K717R^* flies (Figure 5). TIM is not required for PER phosphorylation, but highly phosphorylated PER does not accumulate in *tim^0^* mutants [47]. We observed a small increase of PER molecular weight in *tim^0^ tim>Cul-3^K717R^* flies compared to *tim^0^* controls, indicating that CUL-3 could affect PER in the absence of TIM (Figure 5A, top). The increase in PER molecular weight was slightly higher in a *tim^+^* background, when PER is hypophosphorylated in the control (CT12 and CT36 in Figure 3A and CT12 in Figure 6A). This suggested that TIM could favor the accumulation of phosphorylated PER in *Cul-3* mutants.

As described previously [48], TIM remained mostly unphosphorylated in *per^0^* flies (Figure 5A, bottom). In contrast, *per^0^ tim>Cul-3^K717R^* flies showed accumulation of phosphorylated TIM, thus indicating that CUL-3 does not require PER to induce destabilization of phosphorylated TIM. This result thus supports TIM as being phosphorylated and degraded in *per^0^* flies, in the presence of CUL-3. Interestingly, *per^0^ tim>Cul-3^K717R^* flies had a higher phosphorylated/unphosphorylated TIM ratio than *per^+^ tim>Cul-3^K717R^* flies (see CT 12 in Figures 3A and 6A), suggesting that the presence of PER partly inhibits CUL-3 action on TIM. It has been reported that hypo-phosphorylated TIM is mostly cytoplasmic in *per^0^* flies [49]. Since CUL-3 downregulation affected TIM in the absence of PER, CUL-3 could act in the cytoplasmic compartment. Indeed, TIM immunolabeling was mainly cytoplasmic in both *per^0^* and *per^0^ tim>Cul-3^K717R^* flies, although some faint nuclear labeling was observed in the mutant (Figure 5B). This supported the hypothesis that CUL-3 is able to control the accumulation of phosphorylated TIM in the cytoplasm.

We then asked whether a *tim* deletion affecting TIM phosphorylation and stability would affect CUL-3-dependent stabilization. *tim^0^ tim^Δ260–292^* flies carry a *tim* transgene with a 32 aa deletion in a conserved N-terminal region [50]. This deletion removes a serine-rich region that contains putative phosphorylation sites and TIM^Δ260–292^ migrates faster than TIM in a *per^0^* background on gel electrophoresis [14]. No effect of CUL-3 downregulation could be observed in *tim^0^ tim^Δ260–292^* flies, indicating that the TIM^Δ260–292^ protein is insensitive to CUL-3 control as opposed to the unphosphorylated TIM that accumulates in *per^0^* flies (Figure 5C).

### CUL-3 and SLMB Stabilize Phosphorylated TIM through Different Pathways

In DD, both PER and TIM are stabilized in *slmb^m^* mutants, which do not have detectable SLMB protein [22]. We thus asked whether decreasing CUL-3 activity in *slmb^m^* mutants would show additional effects on TIM stability. In the presence of both PER and TIM (Figure 6A), *slmb^m^* and *tim>Cul-3^K717R^* flies showed a progressive increase of phosphorylated TIM in DD, leading to an equivalent amount of hypo-phosphorylated and phosphorylated protein at day 2 (CT 33 and 39). However, *slmb^m^* mutants showed a slightly higher TIM molecular weight than *tim>Cul-3^K717R^* flies. In the double mutants, a progressive loss of unphosphorylated TIM was observed, leading to a large excess of phosphorylated protein at day 2, migrating similarly to the high molecular weight TIM of *slmb^m^* mutants. Moderately phosphorylated PER accumulated in the *tim>Cul-3^K717R^* flies, whereas hyper-phosphorylated protein was observed in both *slmb^m^* and double mutants, with even higher forms progressively accumulating in the double mutants. To confirm this interaction, PER was analyzed in LD (Figure 6B), where SLMB has limited effects on PER cycling [22]. At ZT3, only a small increase of PER levels was observed in *slmb^m^* and *tim>Cul-3^K717R^* flies, confirming that the downregulation of the two ubiquitin ligases was for a large part compensated by light, although PER was a bit more phosphorylated in the double mutant. At night, moderately phosphorylated PER accumulated in *tim>Cul-3^K717R^* flies, higher forms of phosphorylated PER were observed in *slmb^m^*, and very highly phosphorylated protein accumulated in the double mutant. Our data thus indicated that SLMB and CUL-3 had additive or synergistic effects on PER and TIM phosphorylation. Furthermore, *slmb^m^* mutants appeared to stabilize higher forms of both PER and TIM compared to *tim>Cul-3^K717R^* flies.

To better understand the respective roles of SLMB and CUL-3, we compared the effects of the two mutants on PER and TIM in *tim^0^* and *per^0^* backgrounds, respectively. Whereas *tim^0^ tim>Cul-3^K717R^* flies showed a mild increase of PER phosphorylation, *slmb^m^* (see also [22]) and double mutants accumulated large amounts of similar highly phosphorylated PER when associated with a *tim^0^* mutation (Figure 6C). The absence of TIM thus enhanced the effect of *slmb^m^* mutants on PER, revealing that SLMB-dependent PER degradation is decreased by TIM. Since no difference was observed between *slmb^m^* and the double mutant in a *tim^0^* background, it indicated that CUL-3 requires TIM to show synergistic effects with SLMB on the PER protein. In the absence of PER (Figure 6D), *slmb^m^* mutants accumulated less phosphorylated TIM than in a *per^+^* background, revealing that SLMB-dependent TIM degradation is increased by PER. Since similarly high levels of phosphorylated TIM accumulated in both *per^0^ tim>Cul-3^K717R^* and *per^0^ slmb^m^ tim>Cul-3^K717R^* mutants, it indicated that SLMB requires PER to show additive or synergistic effects with CUL-3 on the TIM protein.

### Different Forms of TIM Make Complexes with CUL-3 and SLMB

**Figure 7:**
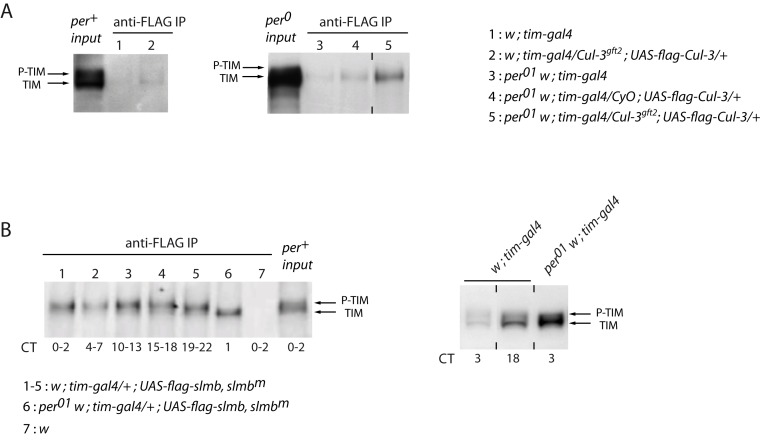
TIM interactions with CUL-3 and SLMB. (A) Anti-CUL-3-FLAG immunoprecipitation from head extracts followed by anti-TIM immunoblotting in *per^+^* (left) and *per^0^* (right) backgrounds. Slots 1 and 3 are negative controls (faint band reveals low non-specific anti-FLAG background), and 50 µg of total proteins were used for *per^+^* and *per^0^* input extracts. Flies were entrained 3 d in LD and transferred to DD for collection at CT15. (B) Left: anti-FLAG-SLMB immunoprecipitation from head extracts collected at different time points, followed by anti-TIM immunoblotting. Two consecutive time points were pooled for IPs in a *per^+^* background. Slot 7 is the negative control, and 50 µg of extracts were used for *per^+^* input. Right: anti-TIM Western blot of head extracts of *w; tim-gal4* (50 µg) and *per^0^ w; tim-gal4* (25 µg) indicate that TIM levels are much higher in a *per^0^* background. At least two independent experiments were done for each assay with very similar results.

Since TIM appears to be the main target of CUL-3, we asked whether the two proteins could be co-immunoprecipitated from head extracts of flies expressing a FLAG-tagged version of CUL-3. A BTB-domain protein is likely to provide the substrate-binding component of the CUL-3 complex and indirect interactions would be predicted. We generated *tim>flag-Cul-3* flies in a heterozygous *Cul-3* mutant background to reduce competition between endogenous and tagged CUL-3 proteins for immunoprecipitation assays. Anti-FLAG immunoprecipitated extracts from such flies showed a faint TIM band that corresponded to hypo-phosphorylated TIM (Figure 7A, left). Since CUL-3 effect on TIM was higher in the absence of PER, a *per^0^* background would be expected to favor TIM-CUL-3 interactions. Indeed, a stronger hypo-phosphorylated TIM band was detected in *per^0^* flies, with TIM amount being further increased in heterozygous *Cul-3^gft2^* mutants (Figure 7A, right). The results thus indicated that CUL-3 and hypo-phosphorylated TIM associate in complexes that are more abundant in the absence of PER. We then compared the properties of TIM/CUL-3 complexes and TIM/SLMB complexes. Immunoprecipitation of tagged SLMB was done in *slmb^m^* flies where tagged SLMB was expressed under *tim-gal4* control to reduce competition between FLAG-SLMB and the endogenous protein. SLMB co-immunoprecipitated more phosphorylated TIM than hypo-phosphorylated TIM in *per^+^* flies, but also co-immunoprecipitated hypo-phosphorylated TIM in a *per^0^* background (Figure 7B, left). However, the amount of TIM/SLMB complexes did not increase in *per^0^* flies, where much higher levels of TIM are observed (Figure 7B, right). In contrast to TIM/CUL-3 complexes, TIM/SLMB complexes thus mostly involve phosphorylated TIM. These complexes are favored in the presence of PER, suggesting that they involve PER-bound TIM.

## Discussion

The control of PER and TIM oscillations in the Drosophila clock relies for a large part on post-translational modifications of the two proteins. PER stability depends on the SLMB-containing SCF ubiquitin ligase complex and requires PER phosphorylation by DBT [11],[22]–[24]. Although TIM stops cycling in *slmb^m^* mutants [22], the circadian control of TIM stability is poorly understood. We have shown here that in addition to SLMB, CUL-3-based ubiquitin ligase complexes play a major role in the control of PER and TIM cycling, with TIM appearing to be the major target of CUL-3 effects. The results indicate that SLMB and CUL-3 differently control the stability of phosphorylated TIM and support opposite effects of PER on the two TIM degradation pathways.

In both LD and DD conditions, *Pdf>Cul-3RNAi* and *tim>Cul-3^K717R^* flies show a nice correlation between dampened PER/TIM oscillations in the PDF neurons and the loss or reduction of morning activity in LD as well as free running rhythms in DD. Since light strongly induces TIM degradation, it likely counteracts the effect of CUL-3 downregulation on TIM cycling and makes difficult the analysis of CUL3 function in LD. The apparently normal TIM oscillations observed in the LNds in LD correlates with the presence of evening activity but contrasts with the dampened oscillations of the sLNvs. It suggests that light-induced TIM degradation is more efficient in the LNds than in the sLNvs, as recently reported in an early night short light pulse paradigm [51], although we cannot exclude that CUL-3 function is more important in the PDF cells. Head extracts of *tim>Cul-3^K717R^* flies in LD also show robust PER and TIM oscillations, but the lower expression of *tim-gal* in the eye likely explains the weaker effects in head extracts compared to PDF cells.

In DD, PER and TIM oscillations are both affected in flies with downregulated CUL-3. However, the dampening of TIM oscillations occurs faster (Figure 3A,B). In addition, limited effects of CUL-3 donwregulation were observed on PER, especially in *tim^0^* flies, whereas a strong effect was observed on TIM and it was increased in a *per^0^* background. Finally, TIM-CUL-3 complexes could be isolated and were more abundant in *per^0^* extracts. These results support TIM as the main target of CUL-3 circadian function, with a major part of the effects on PER being a consequence of TIM modifications. The presence of phosphorylated TIM at all time points in flies expressing dominant negative CUL-3 suggests a post-translational effect of CUL-3 on TIM. *tim>Cul-3^K717R^* flies also show *tim* mRNA changes, but the kinetics of CUL-3 effects on *tim* mRNA levels and TIM protein suggests that the protein is first affected. The strong effect of CUL-3 downregulation on TIM protein in the absence of PER also makes unlikely the possibility that CUL-3 acts on the circadian control of CLK-CYC-dependent transcription. Finally, a decrease of *per* and *tim* mRNA peak levels is the expected consequence of more phosphorylated PER at the end of the day.

Since phosphorylated TIM accumulates in *tim>Cul-3^K717R^* flies, CUL-3 downregulation could either enhance TIM phosphorylation or stabilize phosphorylated TIM. The putative ubiquitin ligase function of CUL-3 rather favors a direct role in the control of TIM levels in the mutants. This would be in agreement with the presence of TIM and CUL-3 in protein complexes, but only the identification of the substrate-recognition protein of the complex will allow us to test for direct interactions. The present data do not exclude that CUL-3 destabilizes an associated TIM kinase. Since no changes in SGG or CK2β levels were observed in *tim>Cul-3^K717R^* flies (unpublished data), more subtle modifications would have to be involved or a yet uncharacterized TIM kinase would have to be destabilized by CUL-3. We thus believe that the simplest interpretation is a direct control of TIM by CUL-3. In this hypothesis, CUL-3 could either control the stability of phosphorylated TIM or control the stability of hypo-phosphorylated TIM, which would then be stabilized and subsequently phosphorylated more extensively. For example, accumulation of phosphorylated PER has been reported in long period *dbt* mutants [5],[52], where defective phosphorylation of the SLMB-binding site induces the subsequent accumulation of normally unstable phosphorylated forms [24]. Why does phosphorylated TIM not accumulate in *Clk>Cul-3RNAi* flies? Since they only show a 2-fold decrease of *Cul-3* mRNA in head extracts, they are likely to display a weak phenotype compared to *tim>Cul-3^K717R^* flies. The absence of phosphorylated TIM in the RNAi flies would thus support CUL-3 acting on several forms of TIM, with phosphorylated TIM being more sensitive to degradation than hypo-phosphorylated TIM.

Even if TIM phosphorylation facilitates CUL-3-controlled degradation, our experiments suggest that CUL-3 preferentially targets TIM proteins that are less phosphorylated than those targeted by SLMB. First, the Co-IP experiments in *per^+^* flies indicate that CUL-3 preferentially binds to hypo-phosphorylated TIM (Figure 7A), whereas SLMB preferentially interacts with phosphorylated TIM (Figure 7B). In addition, TIM-CUL-3 complexes are more abundant in *per^0^* flies, where TIM is hypo-phosphorylated and cytoplasmic, whereas TIM-SLMB complexes are similarly abundant. Second, the absence of PER enhances the effects of CUL-3 on TIM (Figures 5C and 6D), suggesting that CUL-3 preferentially targets unbound TIM protein, which is mostly present in the evening when TIM is less phosphorylated [48]. In contrast, the absence of PER reduced the accumulation of phosphorylated TIM in *slmb^m^* mutants (Figure 6D), suggesting that SLMB preferentially targets PER-bound TIM protein. Most importantly, higher forms of phosphorylated TIM accumulate in *slmb^m^* and double mutants compared to *Cul-3^K717R^* flies (Figure 6A). This suggests that highly phosphorylated TIM is still degraded when CUL-3 is downregulated, while it is not degraded in the absence of SLMB. The results thus favor a model where CUL-3 plays a major role in the control of free less phosphorylated TIM, whereas SLMB appears more important to control PER-bound highly phosphorylated TIM. The accumulation of PER also depends on both TIM-dependent and TIM-independent effects. Both CUL-3 and SLMB play a role in PER degradation in the absence of TIM, but TIM increases CUL-3-controlled degradation, whereas it decreases SLMB-controlled degradation.

CUL-3 complexes recognize substrates through a BTB-domain [53], and the ROADKILL (RDX) protein has been shown to target the CI transcription factor [34],[35]. Expression of several *rdx* RNAi constructs in the clock cells did not show any behavioral phenotype (unpublished data), suggesting that TIM is targeted by another member of the large BTB-domain protein family. A recent study has revealed that the BTB domain protein encoded by the *insomniac* gene is involved in the control of sleep by specifically affecting sleep bout duration, but *insomniac* does not appear to have a circadian function [54]. The importance of serine-rich regions as putative degrons has been revealed for the degradation of CI by the CUL-3/RDX complex [55]. Several serine-rich regions are present in TIM [14] and might thus contain target sites for CUL-3 ubiquitin ligase complexes. The fast migration of TIM^Δ260–292^ in both *Cul-3^+^* and *Cul-3^K717R^* flies suggests that no phosphorylation can occur on this form of TIM or that phosphorylated TIM^Δ260–292^ cannot accumulate even with downregulated CUL-3. The absence of putative phosphorylation sites in TIM^Δ260–292^ [14] may suggest that such sites are required to generate CUL-3-sensitive TIM proteins. At the end of the cycle, TIM is degraded before PER [56], suggesting that TIM degradation may occur within the nuclear PER/TIM complex. Our results rather support SLMB as the major player of this late night nuclear degradation, as it is for PER. SLMB acts on PER through an atypical SLMB-binding site whose progressive phosphorylation during the night increases its affinity for the ubiquitin ligase [24]. No canonical SLMB recognition site was observed in TIM, but the loose conservation of such motifs [24],[57] leaves room for the occurrence of non-canonical sites in the TIM protein. A detailed analysis of TIM phosphorylation sites will be required to understand how the phosphorylation of the protein determines its destabilization by CUL-1 and CUL-3 complexes.

Although both CUL-1 and CUL-3 complexes might control TIM indirectly, the possibility that they share the work to control TIM ubiquitination is interesting. For example, CUL-1/SLMB controls the proteolysis of highly phosphorylated CI, whereas CUL-3/RDX appears to control the degradation of a less phosphorylated form of the protein (see [35]). The preference of CUL-3 for complexing with hypo-phosphorylated TIM and the one of SLMB for targeting more phosphorylated forms suggests that similar mechanisms may apply to TIM. In the mammalian clock, CRY has a function similar to TIM by acting within PER/CRY complexes to repress CLK/BMAL1-dependent transcription [58]. It has been shown that the SLMB ortholog βTrCP controls PER1/2 stability [59]–[61], whereas another F-box ubiquitin ligase, FBXL3, controls CRY stability [62]–[64]. It will be interesting to see whether the two ubiquitin ligases also show preferences for different phosphorylation levels of the clock components.

## Materials and Methods

### Fly Strains and Constructs

The *UAS-Cul-3RNAi* line carries two independent insertions (*CG11861-R1* and *-R2*) of the same RNAi construct, which are described at http://www.shigen.nig.ac.jp/fly/nigfly/index.jsp. The *Cul-3^gft2^* loss-of-function allele has been described in [32], *UAS-Cul-3^ΔC^* in [44], and *UAS-flag-Cul-3* and *UAS-flag-Cul-3^K717R^* in [65]. The *UAS-gfp-Cul-3^K717R^* and *UAS-gfp-Cul-3* have been described in [43], with *UAS-gfp-Cul-3* expression shown to rescue *Cul-3* mutant cellular clones [43]. *Pdf-gal4* [66], *gal1118* [41], *cry-gal4-39* [67], *Clk-gal4-6/1* [40], *tim-gal4-62* [39], *cry-gal80* [38], and *tim^Δ260–292^* [50] flies have been previously reported. *slmb^m^ (slmb^8^ hs-slmb)* adults were produced by providing *hs-slmb* expression with daily heat-shocks during development, as described in [22]. *tim-gal4* and *tim-gal4 UAS-Cul-3^K717R^* flies were in an s-tim background (see [26]). For generating *w;;UAS-flag-slmb* flies, the *slmb* ORF was amplified from cDNA LD08669 (http://flybase.org/reports/FBcl0155568.html) and cloned into the pENTR/D-TOPO vector (Invitrogen). The ORF was recombined into pTFMW (https://dgrc.cgb.indiana.edu/vectors/store/vectors.html) containing a UASt promoter and N-terminal 3xFLAG and 6xMyc tags using LR clonase (Invitrogen). The construct was transformed into *w^1118^* embryos using standard procedures and inserts on chromosomes 2 and 3 were selected. Line 23 was used for the anti-FLAG IP experiments.

### Behavioral Analysis

Experiments were carried out with 1- to 7-d-old males at 25°C, except otherwise indicated, in *Drosophila* activity monitors (TriKinetics). Light was provided by standard, white, fluorescent, low-energy bulbs. Flies were entrained in 12 h∶12 h LD cycles for 4 d and then transferred to DD. Actograms are double-plotted graphs representing the absolute activity levels for each 0.5-h interval, averaged over the total number of flies of a given genotype. LD analysis was done on days 2–4 and DD analysis on days 6–12. Data analysis was done with FaasX software 1.16, which is derived from the Brandeis Rhythm Package (see [68]) and is freely available upon request (Apple Mac OSX only). For LD data, histograms represent the distribution of the activity through 24 h, in 0.5-h intervals, averaged for the total number of flies over three LD cycles. The anticipation phase score is defined as the percentage of averaged activity in the 6 h before the lights-on (or lights-off) transition that occurs in the 3 h before the transition [69]. For DD data, rhythmic flies were defined by χ2 periodogram analysis of a 7 d dataset with the following criteria (filter ON): power ≥20, width ≥2 h, with no selection on period value. Power and width are the height and width of the periodogram peak, respectively, and give the significance of the calculated period. Experiments were reproduced two or three times with very similar results.

### Immunolabelings

Experiments were done on whole-mounted brains as previously described [41]. Primary antibodies were rabbit anti-PER [70] at 1∶15,000, rat anti-TIM [22] at 1∶10,000, and mouse anti-PDF (Developmental Studies Hybridoma Bank) at 1∶50,000. Secondary goat antibodies were FP546-conjugated anti-rabbit (Interchim) at 1∶2,000, Alexa 647–conjugated anti-rat (Molecular Probes) at 1∶5,000, and Alexa 488–conjugated anti-mouse (Molecular Probes) at 1∶2,000. Fluorescence signals were analyzed with a Zeiss AxioImager microscope with an ApoTome structured illumination module. Fluorescence intensity of individual cells was quantified from digital images of single confocal plans with the NIH ImageJ software. We calculated a fluorescence index I = 100n(S−B)/4B, which gives the fluorescence percentage above background (where *n* is the number of labeled cells among the four PDF-expressing s-LNvs, or the three CRY-expressing LNds, S is fluorescence intensity, and B is average intensity of the region adjacent to the positive cell). Index values were then averaged over 12–20 brain hemispheres for each time point.

### Quantitative RT-PCR

Total RNA were prepared from adult heads (about 35) or larvae (3 to 5) using the Promega SV total RNA isolation system. They were quantified using the Nanodrop ND-1000 spectrophotometer and the integrity of the RNA was verified using the Agilent 2100 bioanalyser with the eukaryote total RNA Nano assay. 1 µg of total RNA was reverse-transcribed in a 50 µl final reaction in presence of 0.4 µm oligodT(15), 8 mM dNTP, 40 units of RNasine, and 400 units of M-MLV RTase H-minus (Promega), during 3 h at 37°C. Quantitative PCR was performed with a Roche LightCycler using the SYBR green detection protocol of the manufacturer. 5 µl of a 25 times diluted cDNA were mixed with FastStart DNA master^PLUS^ SYBR green I mix with 500 nM of each primer, the reaction mix was loaded on the capillaries and submitted to 40 cycles of PCR (95°C, 15 s; 60°C, 10 s; 72°C, 20 s), followed by a fusion cycle in order to analyze the melting curve of the PCR products. Negative control without the RTase was introduced to verify the absence of genomic DNA contaminants. Primers (Table S2) were defined within exons using the PrimerSelect program of the Lasergene software (DNAstar). BLAST searches were performed to confirm gene specificity and the absence of multi-locus matching at the primer site. The amplification efficiencies of each pair of primers were generated using the slopes of the standard curves obtained by a four 10-fold dilution series for *tubulin* or an eight 2-fold dilution series for the other genes. All experimental points fell within the linear portion of the curves. The efficiency of the q-PCR amplifications for all pairs of primers is indicated in the table. Amplification specificity for each q-PCR reaction was confirmed by the dissociation curve analysis. Determined Ct values (Table S2) were then used for quantification, with the *tubulin* gene as reference. For each biological replicate (at least two independent samples), measurement was made at least in duplicate (technical replicate).

### Western Blots and Immunoprecipitations

Protein extracts were made from frozen heads homogenized in ice-cold HE extraction buffer (20 mM hepes pH 7.5, 0.1 M KCl, 10 mM EDTA), supplemented with 20 mM glycerophosphate, 0.1 mM DTT, phosphatase inhibitor cocktails 1 (0.5%) and 2 (1%) (Sigma), and protease inhibitor tablets (Roche) according to the manufacturer's instructions. Anti-FLAG immunoprecipitations were performed using EZview Red anti-FLAG affinity gel (Sigma) according to the manufacturer's instructions. For immunoprecipitations, 1 mg of total head proteins were extracted in supplemented HE buffer, and DTT was replaced by 0.1% NP40. For SDS-PAGE, 50 µg of extracts (or immunoprecipitated proteins from 1 mg of extracts) were separated on 3%–8% Tris-acetate (TIM) or 4% Tris-glycine (PER and TIM in Figure 3B) gels (Invitrogen) and transferred to PVDF membranes. Immunoblotting was performed as described previously [22]. Primary antibodies were rabbit anti-PER [70] at 1∶10,000 and rat anti-TIM [22] at 1∶2,000. HRP-conjugated secondary antibodies (Santa Cruz biotechnology) were anti-rabbit goat antibody (1∶40,000) and anti-rat goat antibody (1∶50,000).

## Supporting Information

Figure S1 Locomotor activity of flies expressing *Cul-3RNAi* under *gal1118* control. Flies were entrained for 5 d in LD 12∶12 and then transferred to DD. White and black/gray indicate lights-ON and lights-OFF, respectively. ZT is Zeitgeber Time (ZT0 corresponds to lights-ON). Top panels: averaged activity distribution of *n* flies in LD (see Materials and Methods). Dots indicate the s.e.m. of the activity for each 0.5-h interval. Average activity per 0.5 h is indicated in parentheses on the left. Bottom panels: averaged actograms during both LD and DD conditions (see Materials and Methods). Behavioral analyses were repeated two times with very similar results.
(PDF)

Figure S2 *Cul-3* mRNA levels in flies expressing *Cul-3RNAi*. Quantitative RT-PCR was done as described in Materials and Methods, from either adult heads (top) or L3 larvae (bottom) cDNA with *Cul-3* primers E7-E9. *da-gal4* (*daughterless*) is a ubiquitously expressed *gal4* driver, and *da-gal4/+ ; UAS-Cul-3RNAi/+* flies were lethal after the larval stages. For each experiment, averaged normalized values of the mutant and control genotypes were expressed as a percentage of the maximum value set to 100. Means of three (top) or five (bottom) independent experiments are reported in the graphs. Error bars indicate s.e.m. Two other sets of *Cul-3* primers were tested and gave similar results: *Cul-3* mRNA levels were decreased to 46% (E7-E8 primers) or 45% (E3-E4 primers) of the control levels in *Clk-gal4 ;; UAS-Cul-3-RNAi* heads, and to 28% (E7-E8 primers) or 22% (E3-E4 primers) of the control levels in *da-gal4/+ ; UAS-Cul-3-RNAi/+* larvae.
(PDF)

Figure S3 Locomotor activity of flies expressing *flag-Cul-3^K717R^*, *Cul-3^ΔC^*, or *gfp-Cul-3* transgenes. Flies were entrained for 4 d in LD 12∶12 and then transferred to DD. White and black/gray indicate lights-ON and lights-OFF, respectively. ZT is Zeitgeber Time (ZT0 corresponds to lights-ON). Top panels: averaged activity distribution of *n* flies in LD (see Materials and Methods). Dots indicate the s.e.m. of the activity for each 0.5-h interval. Average activity per 0.5 h is indicated in parentheses on the left. Bottom panels: averaged actograms during both LD and DD conditions (see Materials and Methods). Behavioral analyses were repeated two or three times with very similar results.
(PDF)

Figure S4 Locomotor activity of *Cul-3* downregulated flies at different temperatures. Flies expressing *Cul-3* RNAi and controls were grown at 25°C, and the adults were then either transferred at 20°C or kept at 25°C for 4 d in LD 12∶12 followed by DD. 25°C data are those already shown in Figure 1A. White and black/gray indicate lights-ON and lights-OFF, respectively. ZT is Zeitgeber Time (ZT0 corresponds to lights-ON). Top panels: averaged activity distribution of n flies in LD (see Materials and Methods). Dots indicate the s.e.m. of the activity for each 0.5-h interval. Average activity per 0.5-h is indicated in parentheses on the left. Bottom panels: averaged actograms during both LD and DD conditions (see Materials and Methods). Behavioral analyses were repeated twice with very similar results.
(PDF)

Figure S5 Morphology of PDF-positive s-LNvs in *Cul-3* mutants and controls. Stacks of optical sections from adult brains immunolabeled with anti-PDF. Short arrow indicates slightly more defasciculated projections, and long arrow indicates reduced arborization in the medulla. Flies with a homozygous *tim-gal4* insertion very often show some additional PDF-positive fibers that appear to derive from the Posterior Optic Tract (arrowheads). Scale bars, 50 µM.
(PDF)

Figure S6 TIM immunoreactivity in the LNs of *tim>Cul-3^K717R^* flies. Flies were entrained for 3 d and collected the fourth day of LD at ZT12, 16, or 20. Graphs represent quantifications of TIM immunolabeling in the four “morning” PDF-positive s-LNvs and the three “evening” CRY-positive LNds of *tim>Cul-3^K717R^* flies and controls. Error bars indicate s.e.m. Experiments were repeated twice with very similar results.
(PDF)

Figure S7 TIM Western blot of head extracts of flies expressing CUL-3^K717R^ and controls. Flies were entrained for 3 d in LD and collected every 3 h the fourth day of LD. White and black bars indicate day and night, respectively. ZT is Zeitgeber time. Phosphorylated (P-) and hypo-phosphorylated forms of TIM are indicated. Two independent Western blots were done for each genotype with very similar results.
(PDF)

Figure S8 Quantification of PER and TIM in head extracts of flies expressing CUL-3^K717R^. Phosphorylated (P-) and hypo-phosphorylated forms of PER and TIM were quantified with the Gel Analyzing Tool of Image J software (NIH), which compares the signal density and background of each track. Average values of at least three independent Western blots were used for each genotype/condition. The results are normalized to the maximum value of each blot, set to 100. Error bars indicate s.e.m. Gray and black bars indicate subjective day and subjective night, respectively. CT is circadian time.
(PDF)

Figure S9 TIM protein in head extracts of flies expressing another *Cul-3^K717R^* transgene and controls. Flies were entrained for 3 d in LD and transferred to DD for collection in the second day of DD. Gray and black bars indicate subjective day and subjective night, respectively. CT is circadian time. Phosphorylated (P-) and hypo-phosphorylated forms of TIM are indicated.
(PDF)

Figure S10 Low mobility TIM observed in *tim>Cul-3^K717R^* flies disappears after phosphatase treatment. Flies were entrained for 3 d in LD, transferred to DD, and collected the second day at CT39. Head extracts were treated (+) or not (−) with L-Phosphatase for 30 mn at 30°C and then subjected to electrophoresis and Western Blot analysis with anti-TIM antibodies. Untreated samples stored at 4°C were used to estimate endogenous phosphatase activities, which are not inhibited in the extraction buffer. Phosphatase treatment: protein extracts were made in Lysis Phosphatase buffer (10 mM hepes pH 7.5, 0.1 M KCl, 0.1 mM EDTA, 5% glycerol, 0.1% Triton X-100, 5 mM DTT, 1 mM Mncl2, protease inhibitor tablets) and incubated with 1,000 units of L-phosphatase (New England Biolabs) for 30 mn at 30°C. The experiment was repeated twice with very similar results.
(PDF)

Figure S11 TIM protein in head extracts of flies overexpressing CUL-3 or CUL-3^K717R^ protein and controls. Flies were entrained for 3 d in LD and transferred to DD for collection in the second day of DD. Top: anti-TIM Western blot. Gray and black bars indicate subjective day and subjective night, respectively. CT is circadian time. Phosphorylated (P-) and hypo-phosphorylated forms of TIM are indicated. Two independent experiments were done with similar results for CUL-3 overexpression. A CUL-3^K717R^ blot is shown here for comparison (see Figure 3 for another blot and Figure S8 for quantification). Bottom: quantification of Phosphorylated (P-) and hypo-phosphorylated forms of TIM in flies overexpressing CUL-3 and controls. Bars represent the higher and lower value for each time point in the two independent experiments.
(PDF)

Table S1 Anticipation phase score of fly group activity preceding Lights-ON/Lights-OFF transitions in flies with altered CUL-3 activity. Corresponding activity graphs with activity levels and number of flies are shown in Figures 1, S1, S3, and S4. Anticipation phase score is defined in the Materials and Methods section and is given ± s.e.m. Genotypes with altered CUL-3 activity show no (50%) or low (<60%) morning anticipation compared to controls.
(DOCX)

Table S2 Primers specifications (see Materials and Methods). Efficiency, DNA concentration ratio between cycles *n*+1 and *n* (1<E<2); *R* ^2^, coefficient of determination of the calibration curve; Ct, Cycle threshold. The Ct values interval defines the linear range of the standard curve for each pair of primers.
(DOCX)

## Abbreviations

CI: CUBITUS INTERRUPTUS
CLK: CLOCK
CRY: CRYPTOCHROME
CT: circadian time
CUL-3: CULLIN-3
CYC: CYCLE
DBT: DOUBLE TIME
DD: constant darkness
HH: HEDGEHOG
JET: JETLAG
LD: light-dark
LNds: dorsal lateral neurons
LNs: lateral neurons
PER: PERIOD
RDX: ROADKILL
SGG: SHAGGY
SLMB: SUPERNUMERARY LIMBS
s-LNvs: small ventral lateral neurons
TIM: TIMELESS
ZT: Zeitgeber time
