# Supplementary figures and images for: CULLIN-3 Controls TIMELESS Oscillations in the Drosophila Circadian Clock
Source: PLoS Biol. 2012 Aug 7;10(8):e1001367. doi: 10.1371/journal.pbio.1001367 (PMC3413713; doi:10.1371/journal.pbio.1001367)

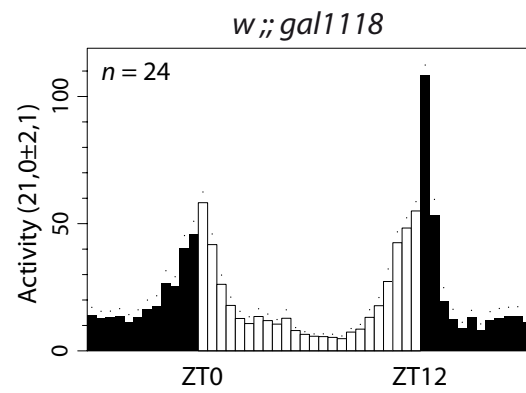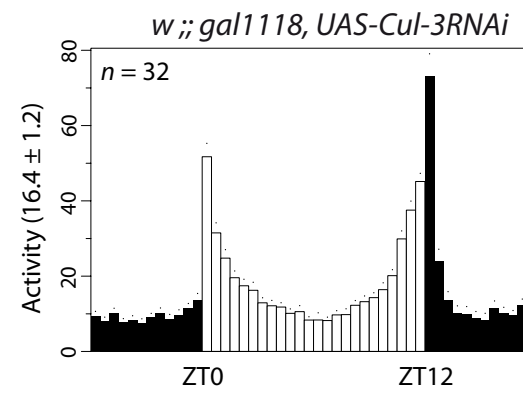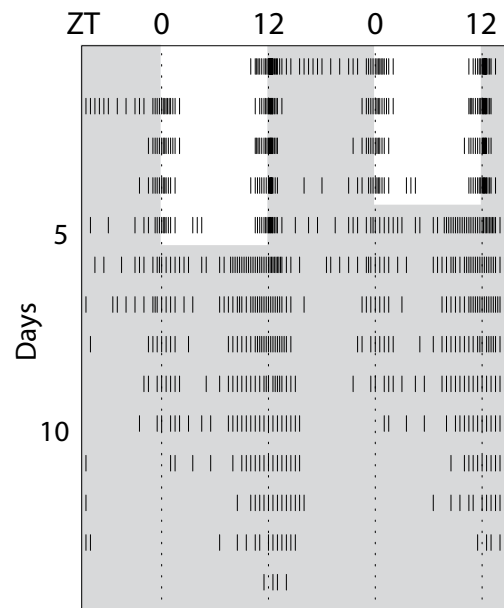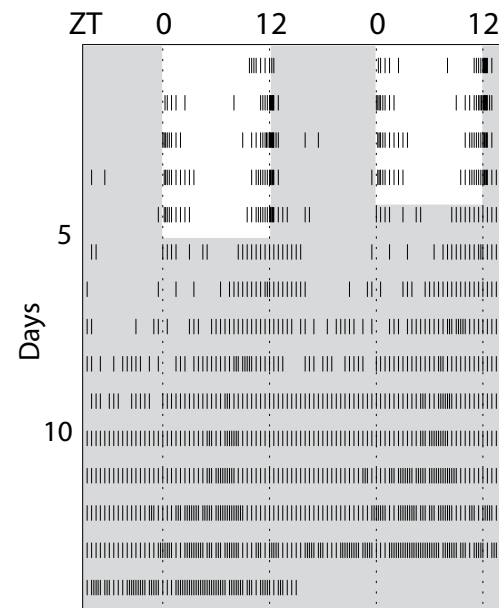

Supplement: Figure S1 — Locomotor activity of flies expressing Cul-3RNAi under gal1118 control. Flies were entrained for 5 d in LD 12∶12 and then transferred to DD. White and black/gray indicate lights-ON and lights-OFF, respectively. ZT is Zeitgeber Time (ZT0 corresponds to lights-ON). Top panels: averaged activity distribution of n flies in LD (see Materials and Methods). Dots indicate the s.e.m. of the activity for each 0.5-h interval. Average activity per 0.5 h is indicated in parentheses on the left. Bottom panels: averaged actograms during both LD and DD conditions (see Materials and Methods). Behavioral analyses were repeated two times with very similar results. (PDF) [file pbio.1001367.s001.pdf]

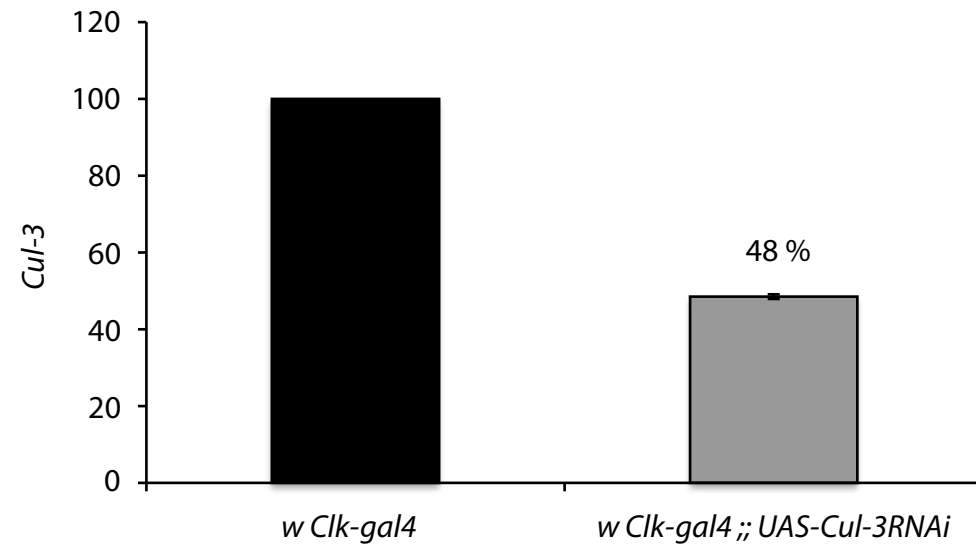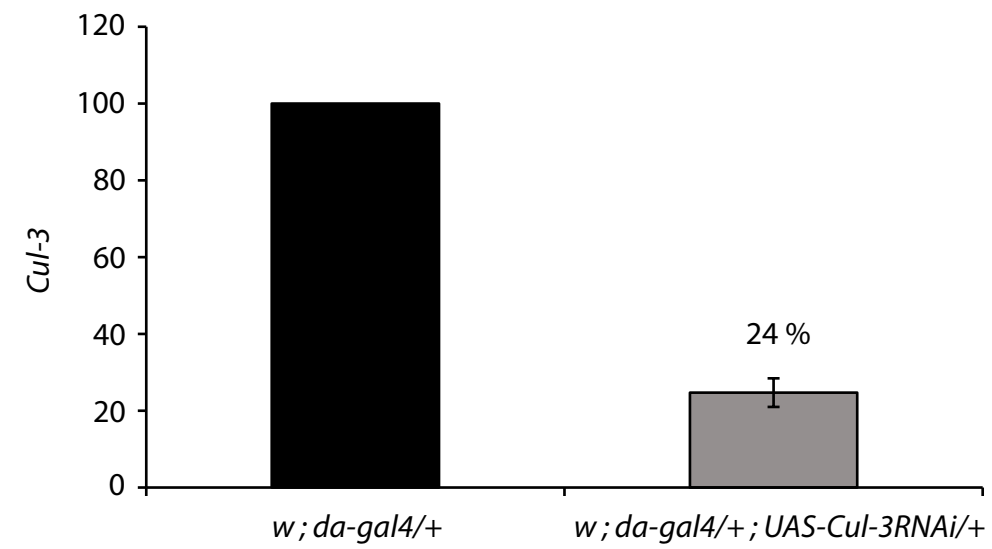

Supplement: Figure S2 — Cul-3 mRNA levels in flies expressing Cul-3RNAi. Quantitative RT-PCR was done as described in Materials and Methods, from either adult heads (top) or L3 larvae (bottom) cDNA with Cul-3 primers E7-E9. da-gal4 (daughterless) is a ubiquitously expressed gal4 driver, and da-gal4/+ ; UAS-Cul-3RNAi/+ flies were lethal after the larval stages. For each experiment, averaged normalized values of the mutant and control genotypes were expressed as a percentage of the maximum value set to 100. Means of three (top) or five (bottom) independent experiments are reported in the graphs. Error bars indicate s.e.m. Two other sets of Cul-3 primers were tested and gave similar results: Cul-3 mRNA levels were decreased to 46% (E7-E8 primers) or 45% (E3-E4 primers) of the control levels in Clk-gal4 ;; UAS-Cul-3-RNAi heads, and to 28% (E7-E8 primers) or 22% (E3-E4 primers) of the control levels in da-gal4/+ ; UAS-Cul-3-RNAi/+ larvae. (PDF) [file pbio.1001367.s002.pdf]

*w; timgal4; UAS-flag-Cul-3<sup>K717R</sup>/+*

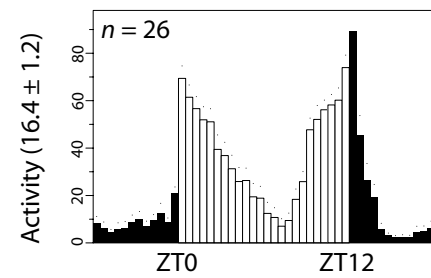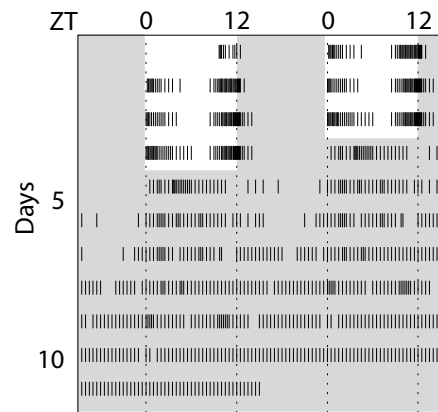

*w; tim-gal4; UAS-Cul-3<sup>ΔC</sup>*

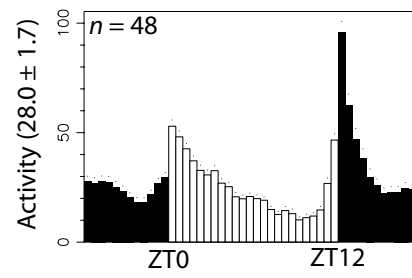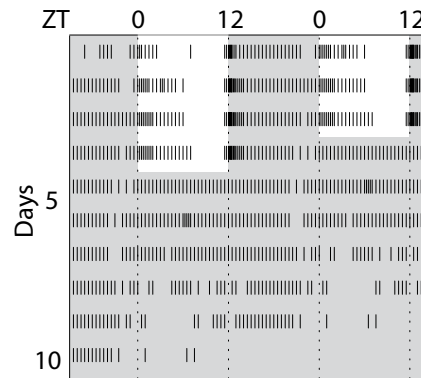

*w; tim-gal4; UAS-gfp-Cul-3*

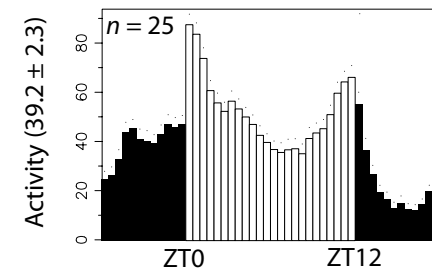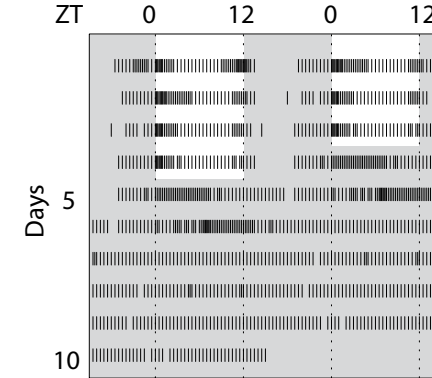

Supplement: Figure S3 — Locomotor activity of flies expressing flag-Cul-3K717R, Cul-3ΔC, or gfp-Cul-3 transgenes. Flies were entrained for 4 d in LD 12∶12 and then transferred to DD. White and black/gray indicate lights-ON and lights-OFF, respectively. ZT is Zeitgeber Time (ZT0 corresponds to lights-ON). Top panels: averaged activity distribution of n flies in LD (see Materials and Methods). Dots indicate the s.e.m. of the activity for each 0.5-h interval. Average activity per 0.5 h is indicated in parentheses on the left. Bottom panels: averaged actograms during both LD and DD conditions (see Materials and Methods). Behavioral analyses were repeated two or three times with very similar results. (PDF) [file pbio.1001367.s003.pdf]

*w; Pdf-gal4*  
(20°C)

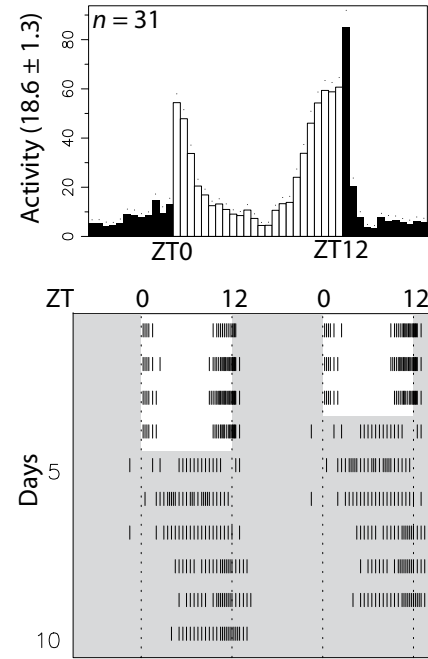

*w; Pdf-gal4*  
(25°C)

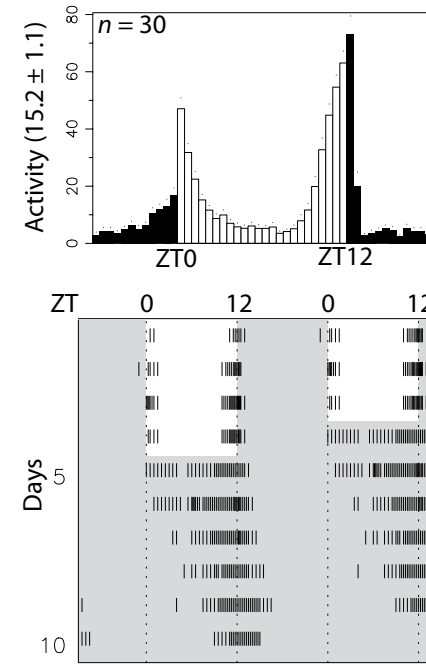

*w; Pdf-gal4; UAS-Cul-3RNAi*  
(20°C)

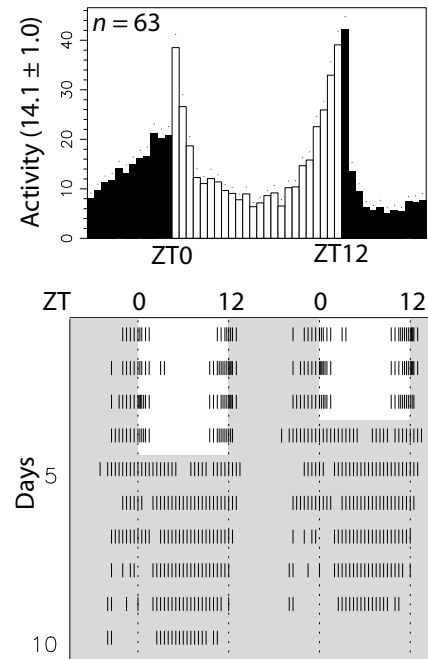

*w; Pdf-gal4; UAS-Cul-3RNAi*  
(25°C)

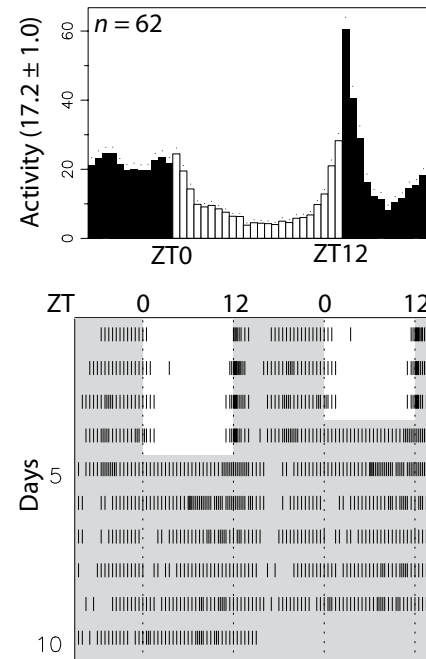

Supplement: Figure S4 — Locomotor activity of Cul-3 downregulated flies at different temperatures. Flies expressing Cul-3 RNAi and controls were grown at 25°C, and the adults were then either transferred at 20°C or kept at 25°C for 4 d in LD 12∶12 followed by DD. 25°C data are those already shown in Figure 1A. White and black/gray indicate lights-ON and lights-OFF, respectively. ZT is Zeitgeber Time (ZT0 corresponds to lights-ON). Top panels: averaged activity distribution of n flies in LD (see Materials and Methods). Dots indicate the s.e.m. of the activity for each 0.5-h interval. Average activity per 0.5-h is indicated in parentheses on the left. Bottom panels: averaged actograms during both LD and DD conditions (see Materials and Methods). Behavioral analyses were repeated twice with very similar results. (PDF) [file pbio.1001367.s004.pdf]

TIM

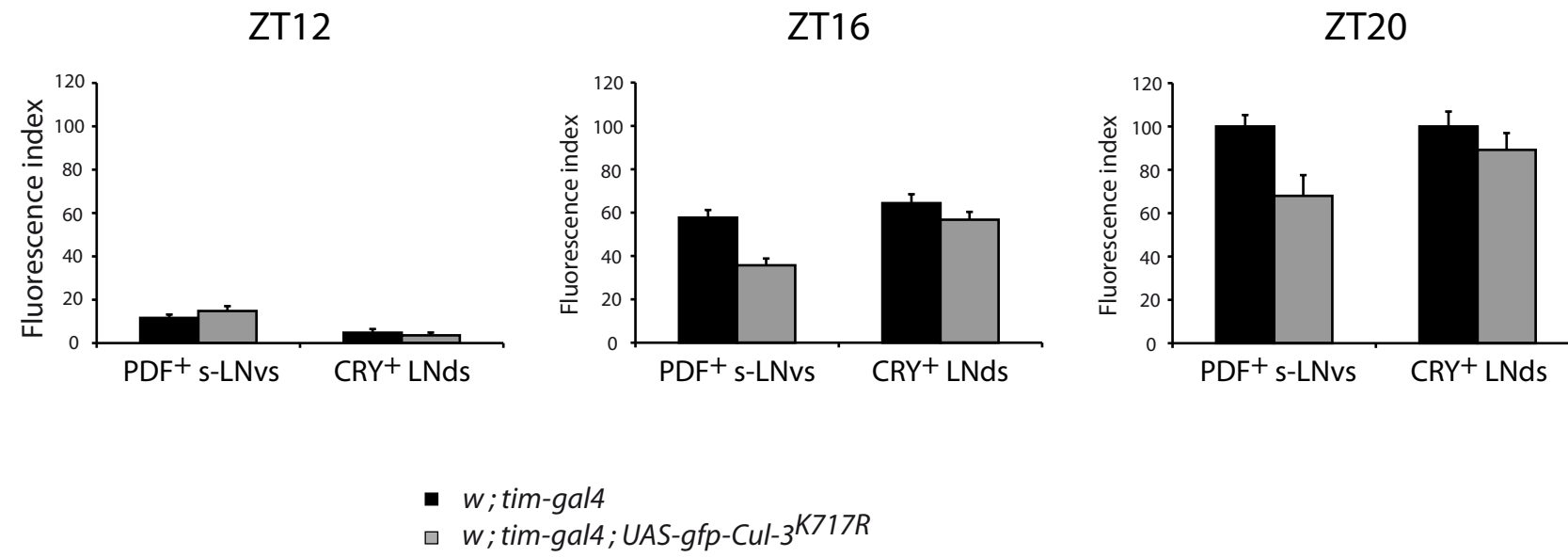

Supplement: Figure S6 — TIM immunoreactivity in the LNs of tim>Cul-3K717R flies. Flies were entrained for 3 d and collected the fourth day of LD at ZT12, 16, or 20. Graphs represent quantifications of TIM immunolabeling in the four “morning” PDF-positive s-LNvs and the three “evening” CRY-positive LNds of tim>Cul-3K717R flies and controls. Error bars indicate s.e.m. Experiments were repeated twice with very similar results. (PDF) [file pbio.1001367.s006.pdf]

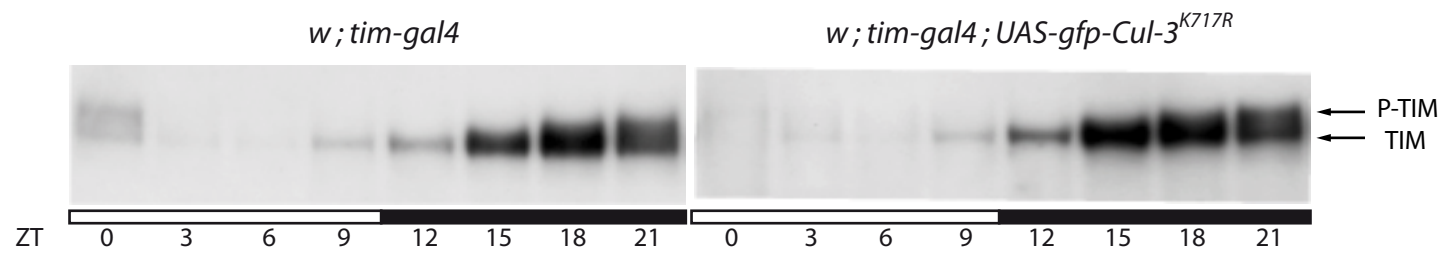

Supplement: Figure S7 — TIM Western blot of head extracts of flies expressing CUL-3K717R and controls. Flies were entrained for 3 d in LD and collected every 3 h the fourth day of LD. White and black bars indicate day and night, respectively. ZT is Zeitgeber time. Phosphorylated (P-) and hypo-phosphorylated forms of TIM are indicated. Two independent Western blots were done for each genotype with very similar results. (PDF) [file pbio.1001367.s007.pdf]

PER

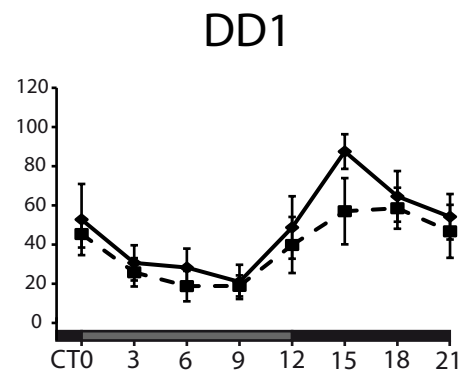

DD2

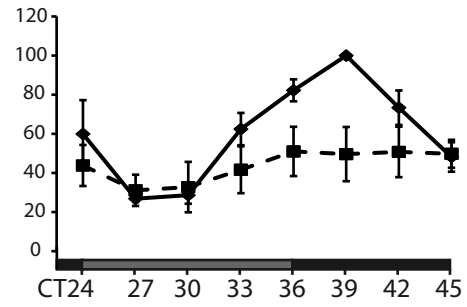

P-PER

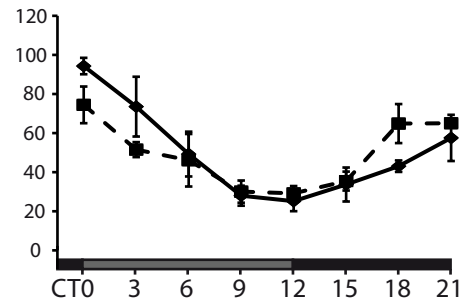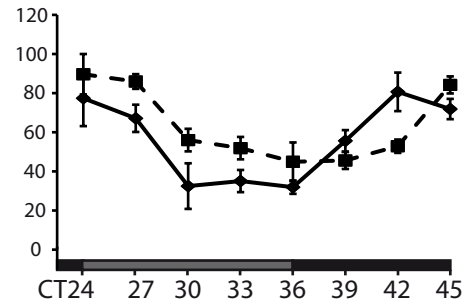

TIM

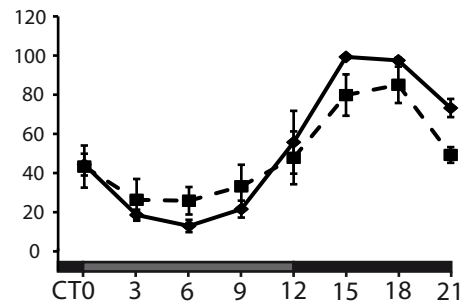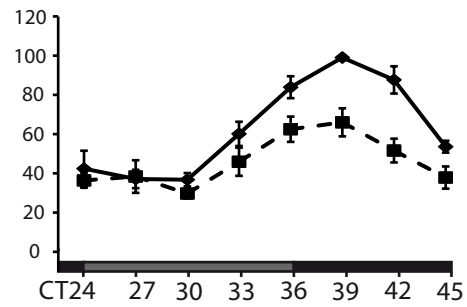

P-TIM

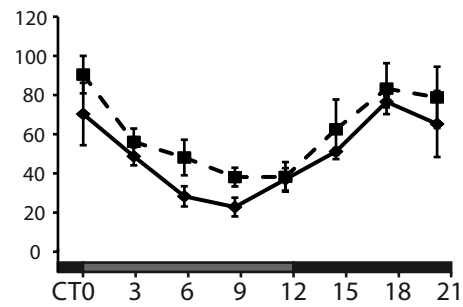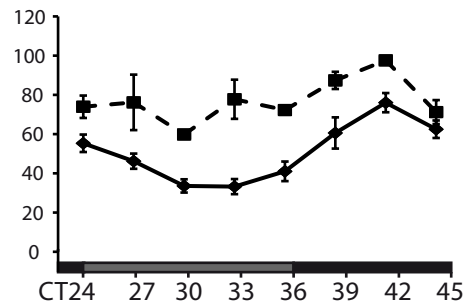

—●— *w;tim-gal4*  
 -■- *w;tim-gal4;UAS-gfp-Cul-3<sup>K717R</sup>*

Supplement: Figure S8 — Quantification of PER and TIM in head extracts of flies expressing CUL-3K717R. Phosphorylated (P-) and hypo-phosphorylated forms of PER and TIM were quantified with the Gel Analyzing Tool of Image J software (NIH), which compares the signal density and background of each track. Average values of at least three independent Western blots were used for each genotype/condition. The results are normalized to the maximum value of each blot, set to 100. Error bars indicate s.e.m. Gray and black bars indicate subjective day and subjective night, respectively. CT is circadian time. (PDF) [file pbio.1001367.s008.pdf]

*w; tim-gal4*

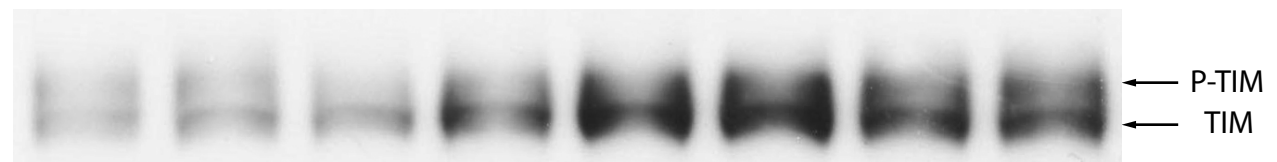

*w; tim-gal4; UAS-flag-Cul-3<sup>K717R</sup>/+*

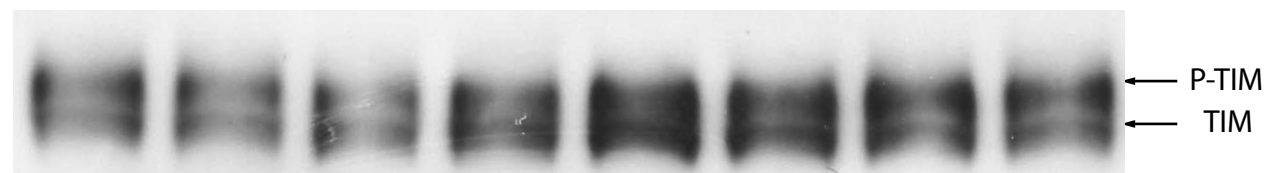

CT24 27 30 33 36 39 42 45

Supplement: Figure S9 — TIM protein in head extracts of flies expressing another Cul-3K717R transgene and controls. Flies were entrained for 3 d in LD and transferred to DD for collection in the second day of DD. Gray and black bars indicate subjective day and subjective night, respectively. CT is circadian time. Phosphorylated (P-) and hypo-phosphorylated forms of TIM are indicated. (PDF) [file pbio.1001367.s009.pdf]

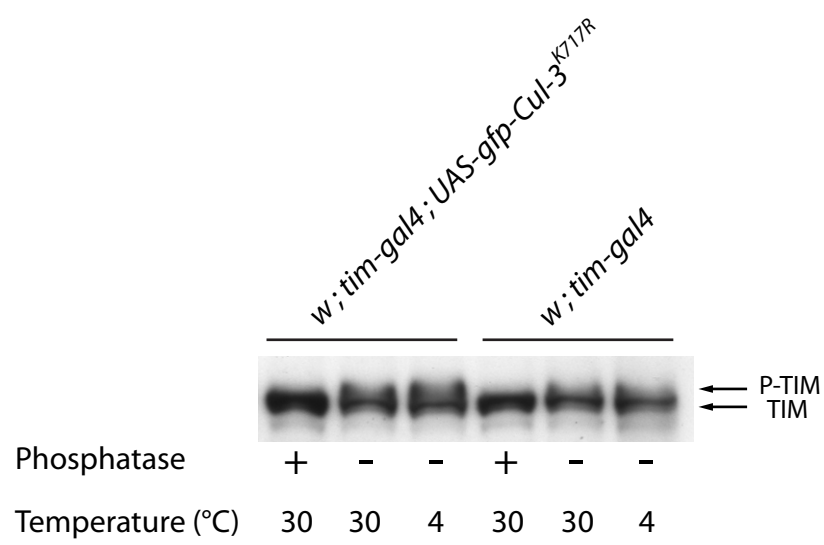

Supplement: Figure S10 — Low mobility TIM observed in tim>Cul-3K717R flies disappears after phosphatase treatment. Flies were entrained for 3 d in LD, transferred to DD, and collected the second day at CT39. Head extracts were treated (+) or not (−) with L-Phosphatase for 30 mn at 30°C and then subjected to electrophoresis and Western Blot analysis with anti-TIM antibodies. Untreated samples stored at 4°C were used to estimate endogenous phosphatase activities, which are not inhibited in the extraction buffer. Phosphatase treatment: protein extracts were made in Lysis Phosphatase buffer (10 mM hepes pH 7.5, 0.1 M KCl, 0.1 mM EDTA, 5% glycerol, 0.1% Triton X-100, 5 mM DTT, 1 mM Mncl2, protease inhibitor tablets) and incubated with 1,000 units of L-phosphatase (New England Biolabs) for 30 mn at 30°C. The experiment was repeated twice with very similar results. (PDF) [file pbio.1001367.s010.pdf]

*w; tim-gal4*

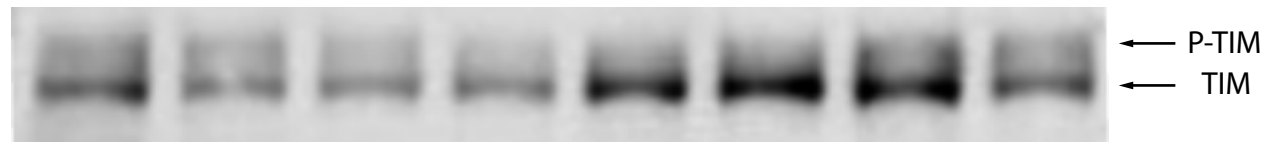

*w; tim-gal4; UAS-gfp-Cul-3<sup>K717R</sup>*

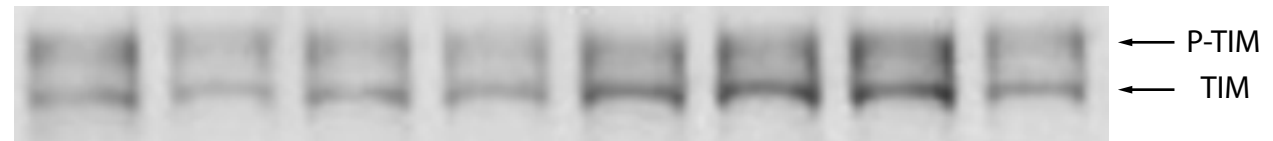

*w; tim-gal4; UAS-gfp-Cul-3*

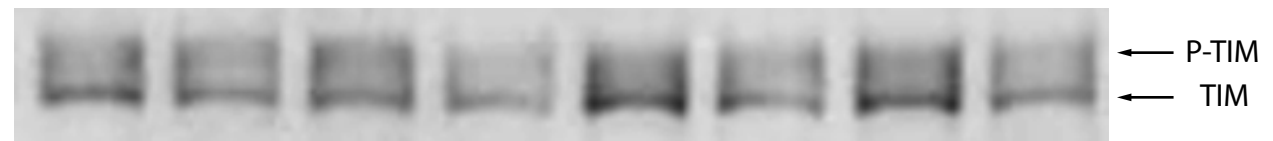

CT24 27 30 33 36 39 42 45

TIM

—■— *w; tim-gal4*  
—▲— *w; tim-gal4; UAS-gfp-Cul-3*

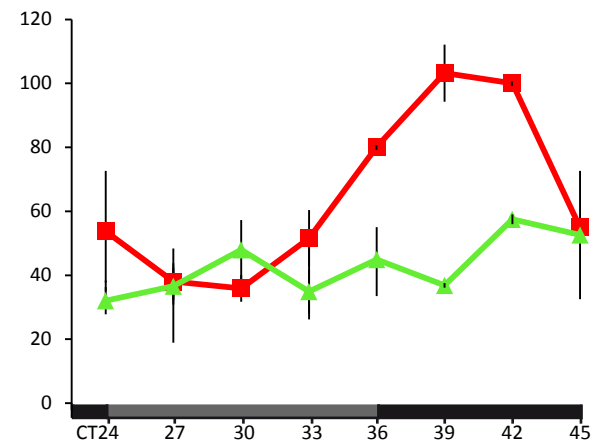

P-TIM

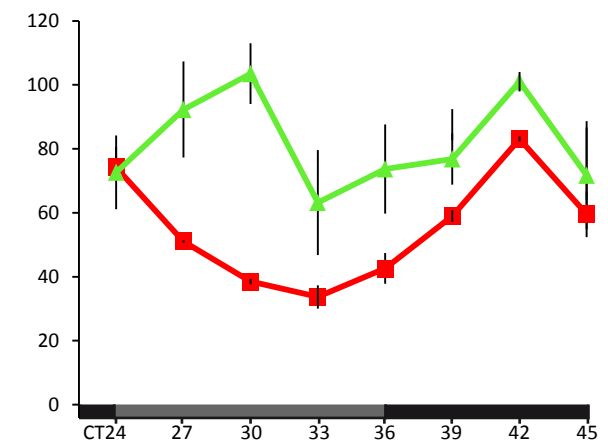

Supplement: Figure S11 — TIM protein in head extracts of flies overexpressing CUL-3 or CUL-3K717R protein and controls. Flies were entrained for 3 d in LD and transferred to DD for collection in the second day of DD. Top: anti-TIM Western blot. Gray and black bars indicate subjective day and subjective night, respectively. CT is circadian time. Phosphorylated (P-) and hypo-phosphorylated forms of TIM are indicated. Two independent experiments were done with similar results for CUL-3 overexpression. A CUL-3K717R blot is shown here for comparison (see Figure 3 for another blot and Figure S8 for quantification). Bottom: quantification of Phosphorylated (P-) and hypo-phosphorylated forms of TIM in flies overexpressing CUL-3 and controls. Bars represent the higher and lower value for each time point in the two independent experiments. (PDF) [file pbio.1001367.s011.pdf]
