# Supplementary material for: CULLIN-3 Controls TIMELESS Oscillations in the Drosophila Circadian Clock
Source: PLoS Biol. 2012 Aug 7;10(8):e1001367. doi: 10.1371/journal.pbio.1001367 (PMC3413713; doi:10.1371/journal.pbio.1001367)
Supplement: Figure S5 — Morphology of PDF-positive s-LNvs in Cul-3 mutants and controls. Stacks of optical sections from adult brains immunolabeled with anti-PDF. Short arrow indicates slightly more defasciculated projections, and long arrow indicates reduced arborization in the medulla. Flies with a homozygous tim-gal4 insertion very often show some additional PDF-positive fibers that appear to derive from the Posterior Optic Tract (arrowheads). Scale bars, 50 µM. (PDF) [file pbio.1001367.s005.pdf]

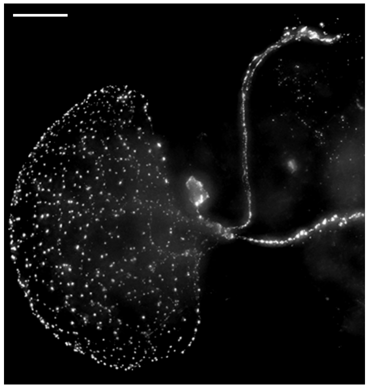

w ; Pdf-gal4

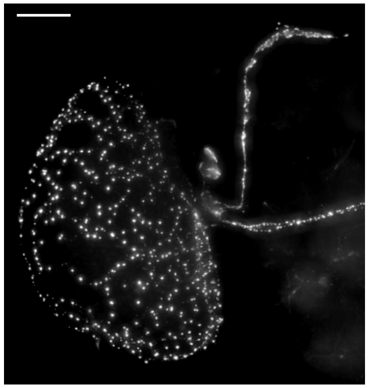

w ; Pdf-gal4 ; UAS-Cul-3RNAi

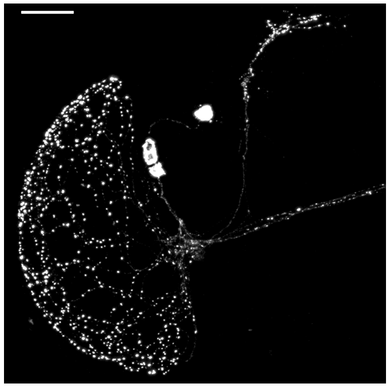

w ;; gal1118

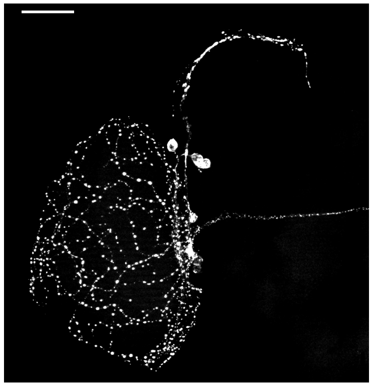

w ;; gal1118, UAS-Cul-3RNAi

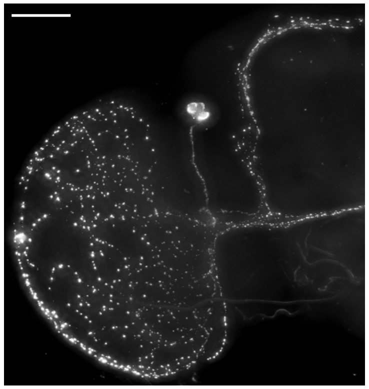

w ; tim-gal4

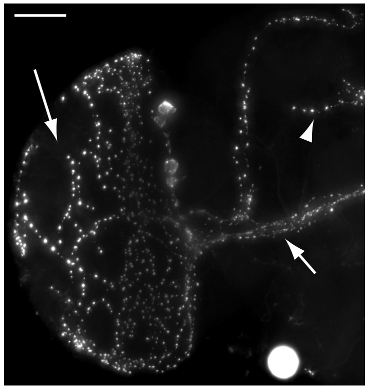

w ; tim-gal4 ; UAS-Cul-3$^{\Delta C}$

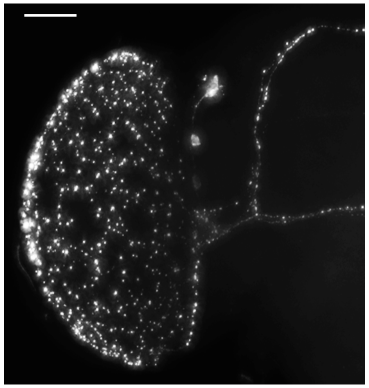

w ; tim-gal4 ; UAS-gfp-Cul-3^{K717R}

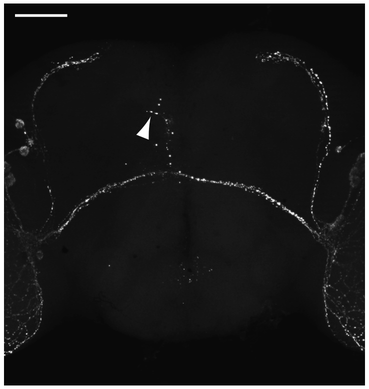

w ; tim-gal4

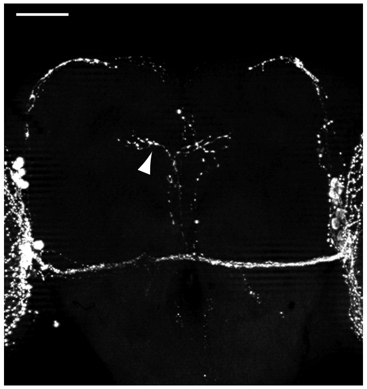

w ; tim-gal4 ; UAS-Cul-3$^{\Delta C}$
