# Supplementary material for: CULLIN-3 Controls TIMELESS Oscillations in the Drosophila Circadian Clock
Source: PLoS Biol. 2012 Aug 7;10(8):e1001367. doi: 10.1371/journal.pbio.1001367 (PMC3413713; doi:10.1371/journal.pbio.1001367)
Supplement: Table S1 — Anticipation phase score of fly group activity preceding Lights-ON/Lights-OFF transitions in flies with altered CUL-3 activity. Corresponding activity graphs with activity levels and number of flies are shown in Figures 1, S1, S3, and S4. Anticipation phase score is defined in the Materials and Methods section and is given ± s.e.m. Genotypes with altered CUL-3 activity show no (50%) or low (<60%) morning anticipation compared to controls. (DOCX) [file pbio.1001367.s012.docx]

Table S1: Anticipation phase score of fly group activity preceding Lights-ON/ Lights-OFF transitions in flies with altered CUL-3 activity.

| Genotype | Morning Anticipation Phase Score (%) | Evening Anticipation Phase Score (%) |
| --- | --- | --- |
| *w ; Pdf-gal4 ; UAS-Cul-3RNAi* | 49.6 ± 0.01 | 76.7 ± 0.02 |
| *w ; Pdf-gal4* | 69.7 ± 0.04 | 87.4 ± 0.02 |
|  |  |  |
| *w Clk-gal4 ;; UAS-Cul-3RNAi* | 53.2 ± 0.03 | 64.6 ± 0.02 |
| *w Clk-gal4* | 69.5 ± 0.08 | 78.8 ± 0.02 |
|  |  |  |
| *w ;; gal1118, UAS-Cul-3RNAi* | 55.0 ± 0.01 | 72.9 ± 0.01 |
| *w ;; gal1118* | 70.1 ± 0.02 | 84.4 ± 0.01 |
|  |  |  |
| *w ;; gal1118,* *UAS-gfp-Cul-3^K717R^* | 57.8 ± 0.05 | 66.3 ± 0.01 |
| *w ;; gal1118* | 65.7 ± 0.02 | 65.3 ± 0.02 |
|  |  |  |
| *w ; tim-gal4 ;* *UAS-gfp-Cul-3^K717R^* | 50.1 ± 0.01 | 60.6 ± 0.01 |
| *w ; tim-gal4 ; UAS-flag-Cul-3^K717R^/+* | 59.1 ± 0.05 | 82.9 ± 0.03 |
| *w ; tim-gal4 ; UAS-Cul-3^∆C^* | 47.4 ± 0.02 | 57.8 ± 0.02 |
| *w ; tim-gal4 ; UAS-gfp-Cul-3* | 56.6 ± 0.02 | 59.0 ± 0.02 |
| *w ; tim-gal4* | 68.4 ± 0.01 | 64.0 ± 0.01 |
|  |  |  |
| *w ; Pdf-gal4 ; UAS-Cul-3RNAi (20°C)* | 63.0 ± 0.02 | 75.1 ± 0.01 |
| *w ; Pdf-gal4 (20°C)* | 67.2 ± 0.04 | 81.8 ± 0.02 |

Corresponding activity graphs with activity levels and number of flies are shown in Figures 1, S1, S3 and S4. Anticipation phase score is defined in the Material and Methods section, and is given ± s.e.m. Genotypes with altered CUL-3 activity show no (50%) or low (< 60%) morning anticipation compared to controls.
