# Supplementary material for: CULLIN-3 Controls TIMELESS Oscillations in the Drosophila Circadian Clock
Source: PLoS Biol. 2012 Aug 7;10(8):e1001367. doi: 10.1371/journal.pbio.1001367 (PMC3413713; doi:10.1371/journal.pbio.1001367)
Supplement: Table S2 — Primers specifications (see Materials and Methods). Efficiency, DNA concentration ratio between cycles n+1 and n (1<E<2); R 2, coefficient of determination of the calibration curve; Ct, Cycle threshold. The Ct values interval defines the linear range of the standard curve for each pair of primers. (DOCX) [file pbio.1001367.s013.docx]

Table S2: Primers specifications (see Material and Methods).

| Gene (exons) | 5' primer | 3' primer | Amplicon size | Efficiency | R^2^ | Ct |
| --- | --- | --- | --- | --- | --- | --- |
|  |  |  |  |  |  |  |
| *tubulin* (E1-E2) | TCCTTGTCGCGTGTGAAACA | CCGAACGAGTGGAAGATGAG | 464 bp | 1.90 | 0.99 | 17-28 |
|  |  |  |  |  |  |  |
| *period* (E6-E7) | ACCCGCATCCTTCGCTTTTCTACA | AGGTGAGCCAGTGGTGAGGACGGG | 219 bp | 1.89 | 0.99 | 21.5-27.5 |
|  |  |  |  |  |  |  |
| *timeless* (E12-E13) | AGTTGGTCATGCGCAGCAAATG | TCCTTTTCGTACACAGATGCCA | 447 bp | 1.90 | 0.99 | 19-25 |
|  |  |  |  |  |  |  |
| *Cullin-3* (E7-E9) | AAAGATGCACCCAGCTCCAGTTCA | GCCGCTTCGATCTCGTGCTTAC | 410 bp | 1.91 | 0.99 | 22-28 |
|  |  |  |  |  |  |  |
| *Cullin-3* (E7-E8) | TAAAGATGCACCCAGCTCCAGTTC | GGCTCAATGTCCTTAGTTTTCGTT | 261 bp | 1.89 | 0.99 | 22-28 |
|  |  |  |  |  |  |  |
| *Cullin-3* (E3-E4) | GGACGCATGCTGGTTAAGGAAGAA | CCAATAGGAAACGGAAGAGAACCA | 301 bp | 1.90 | 0.99 | 22-28 |

Efficiency: DNA concentration ratio between cycles n+1 and n (1 < E < 2)

R^2^: coefficient of determination of the calibration curve.

Ct: Cycle threshold. The Ct values interval defines the linear range of the standard curve for each pair of primers
